# Gene Structure-Based Homology Search Identifies Highly Divergent Putative Effector Gene Family

**DOI:** 10.1093/gbe/evac069

**Published:** 2022-05-09

**Authors:** David L. Stern, Clair Han

**Affiliations:** Janelia Research Campus of the Howard Hughes Medical Institute, 19700 Helix Drive, Ashburn, VA 20147, USA; Janelia Research Campus of the Howard Hughes Medical Institute, 19700 Helix Drive, Ashburn, VA 20147, USA

**Keywords:** gene homology, gene structure, genome evolution, rapidly evolving genes, effector genes, aphid gall

## Abstract

Homology of highly divergent genes often cannot be determined from sequence similarity alone. For example, we recently identified in the aphid *Hormaphis cornu* a family of rapidly evolving *bicycle* genes, which encode novel proteins implicated as plant gall effectors, and sequence similarity search methods yielded few putative *bicycle* homologs in other species. Coding sequence-independent features of genes, such as intron-exon boundaries, often evolve more slowly than coding sequences, however, and can provide complementary evidence for homology. We found that a linear logistic regression classifier using only structural features of *bicycle* genes identified many putative *bicycle* homologs in other species. Independent evidence from sequence features and intron locations supported homology assignments. To test the potential roles of *bicycle* genes in other aphids, we sequenced the genome of a second gall-forming aphid, *Tetraneura nigriabdominalis* and found that many *bicycle* genes are strongly expressed in the salivary glands of the gall forming foundress. In addition, *bicycle* genes are strongly overexpressed in the salivary glands of a non-gall forming aphid, *Acyrthosiphon pisum*, and in the non-gall forming generations of *H. cornu*. These observations suggest that Bicycle proteins may be used by multiple aphid species to manipulate plants in diverse ways. Incorporation of gene structural features into sequence search algorithms may aid identification of deeply divergent homologs, especially of rapidly evolving genes involved in host-parasite interactions.

Significance

Many related genes, and especially genes involved in host-parasite interactions, have changed so extensively between species that similarities in the primary amino acid sequence are no longer detectable by standard search methods. The structure of such genes, however, such as the lengths of exons and the positions of introns, often evolves more slowly than the gene sequences themselves. We have exploited this relatively slow evolution of gene structure to identify a large family of rapidly evolving genes that are expressed in the salivary glands of plant sap-sucking insects and that may facilitate their ability to parasitize plants.

## Introduction

One challenge that is faced by evolutionary and functional genetic studies is that many genes have been identified as “orphan” or lineage-specific genes because homologous sequences cannot be identified outside of a limited taxonomic range. Many lineage-specific genes may not be truly novel, however, since sequence divergence can cause homologs to become undetectable by sequence-search methods (Vakirlis et al., 2020; Weisman et al., 2020). Identifying such extremely divergent homologs remains a significant bioinformatic challenge and limits functional inferences derived from homology (Loewenstein et al., 2009).

Genes involved in host-parasite systems often evolve extremely divergent sequences as a result of genetic conflict (Paterson et al., 2010; Eizaguirre et al., 2012). Aphids and their host plants represent one such antagonistic pair. Aphids are small insects that feed by inserting their thin mouthparts (stylets) into the phloem vessels of plants to extract nutrients (Dixon, 1978). Like many herbivorous insects, aphids introduce effector molecules into plant tissues to manipulate the physiology and development of plants to the insects’ advantage (Mutti et al., 2008; Hogenhout and Bos, 2011; Elzinga and Jander, 2013; Rodriguez et al., 2017). For example, aphids introduce calcium binding proteins that prevent the plant's ability to block phloem cell transport in response to phloem vessel damage (Will et al., 2007). It is believed that aphids introduce a wide range of effector proteins and that these molecules contribute to the debilitating effects of multiple aphid species on plants, which imposes significant financial damage on most major agricultural crops worldwide (Schaible et al., 2008).

In an extreme form of aphid manipulation of plant physiology and development, some aphid species induce novel plant organs, called galls, which provide the aphids with a ready food source and with protection from the elements and from natural enemies (Shorthouse et al., 2005; Giron et al., 2016). Aphid galls can be significant nutrient sinks (Burstein et al., 1994), demonstrating that galling aphids induce both local and long-range changes to plant physiology.

Many arthropods induce galls on a wide diversity of plant species (Mani, 1964), but the complex life cycle of galling aphids provides unique advantages for studying the mechanisms of gall induction that are not found in other galling arthropods. In most galling aphid species, only one generation each year, the gall foundress or fundatrix, appears to be capable of inducing galls. Individuals of subsequent generations in the life cycle do not induce galls, even though they may feed on the same host plant. Comparisons of genes transcribed specifically in salivary glands of individuals across generations provided some of the first clues to the putative effector proteins contributing to gall induction (Korgaonkar et al., 2021).

We reported recently that the genome of one species of galling aphid encodes 476 related genes encoding diverse presumptive effector proteins with no sequence homology to previously described proteins and that many of these proteins contain two cysteine-tyrosine-cysteine (CYC) motifs (Korgaonkar et al., 2021). These genes were therefore named *bicycle* (bi-CYC-like) genes. A genome-wide association study revealed that variation in one *bicycle* gene, called *determinant of gall color* (*dgc*), was strongly associated with a red versus green gall color polymorphism and this genetic polymorphism was associated with strong downregulation of *dgc* in aphids and strong upregulation of a small number of genes involved in anthocyanin production in plant galls. In addition, the majority of *bicycle* genes are strongly upregulated in the salivary glands specifically of the aphid generation that induces galls (the fundatrix). Together with genetic evidence that *dgc* regulates the gall phenotype, the enrichment of *bicycle* genes expressed in fundatrix salivary glands suggested that many *bicycle* genes contribute to gall development or physiology.

The primary amino-acid sequences of Bicycle proteins have evolved rapidly, apparently in response to extremely strong positive selection (Korgaonkar et al., 2021). In preliminary studies, we attempted to identify *bicycle* homologs in other insect species using sequence similarity algorithms such as *Basic Local Alignment Search Tool (BLAST)* (Altschul et al., 1990) and *hmmer* (Johnson et al., 2010; Eddy, 2011), but identified few putative homologs. It was therefore unclear if *bicycle* genes are a recently evolved family of genes or an ancient family that has evolved highly divergent coding sequences.

Extremely divergent homologs have been identified previously using features of gene structures that evolve more slowly than the coding sequence, such as exon sizes and intron positions (Bazan, 1991; Brown et al., 1995; Betts, 2001), and these characteristics of genes have also proven to be valuable for phylogeny reconstruction (Rokas et al., 1999; Venkatesh et al., 1999; Rokas and Holland, 2000; Telford and Copley, 2011). We noted previously that *bicycle* genes have unusual gene structures containing many micro-exons (Korgaonkar et al., 2021), and we considered the possibility that *bicycle* gene structure may be more conserved than *bicycle* gene sequences. To explore the evolutionary history of *bicycle* genes, we therefore sought a method that would allow identification of highly divergent *bicycle* gene homologs that did not rely on sequence similarity. To accomplish this, we built a logistic regression classifier based only on structural features of *bicycle* genes. We found that this classifier was very accurate, despite the fact that it does not include any sequence information. This classifier identified many highly-divergent *bicycle* homologs in *Hormaphis cornu* that could not be identified by sequence similarity search methods, including genes that encode proteins that do not possess the previously canonical CYC motif. In addition, the classifier identified many highly divergent candidate *bicycle* homologs in all aphids, phylloxerans, and scale insects we studied. We did not detect any putative homologs in three progressively basal outgroups. Multiple sequence alignment of these putative homologs revealed that many contain N-terminal signal sequences and CYC motifs, consistent with their assignment as *bicycle* homologs. In addition, gene-structure aware sequence alignment revealed multiple apparently shared intron boundaries between putative homologs that share little obvious sequence similarity. In addition, putative *bicycle* homologs are highly enriched in the salivary gland mRNA of two gall forming and one non-gall forming aphid. All of this evidence supports the hypothesis that *bicycle* gene homologs encode effector proteins in gall-forming and non-gall-forming aphids and also in phylloxerans and scale insects.

## Results

### Sequence-Based Homology Searches Find Few Bicycle Genes Outside of *H. cornu*

**Fig. 1.**
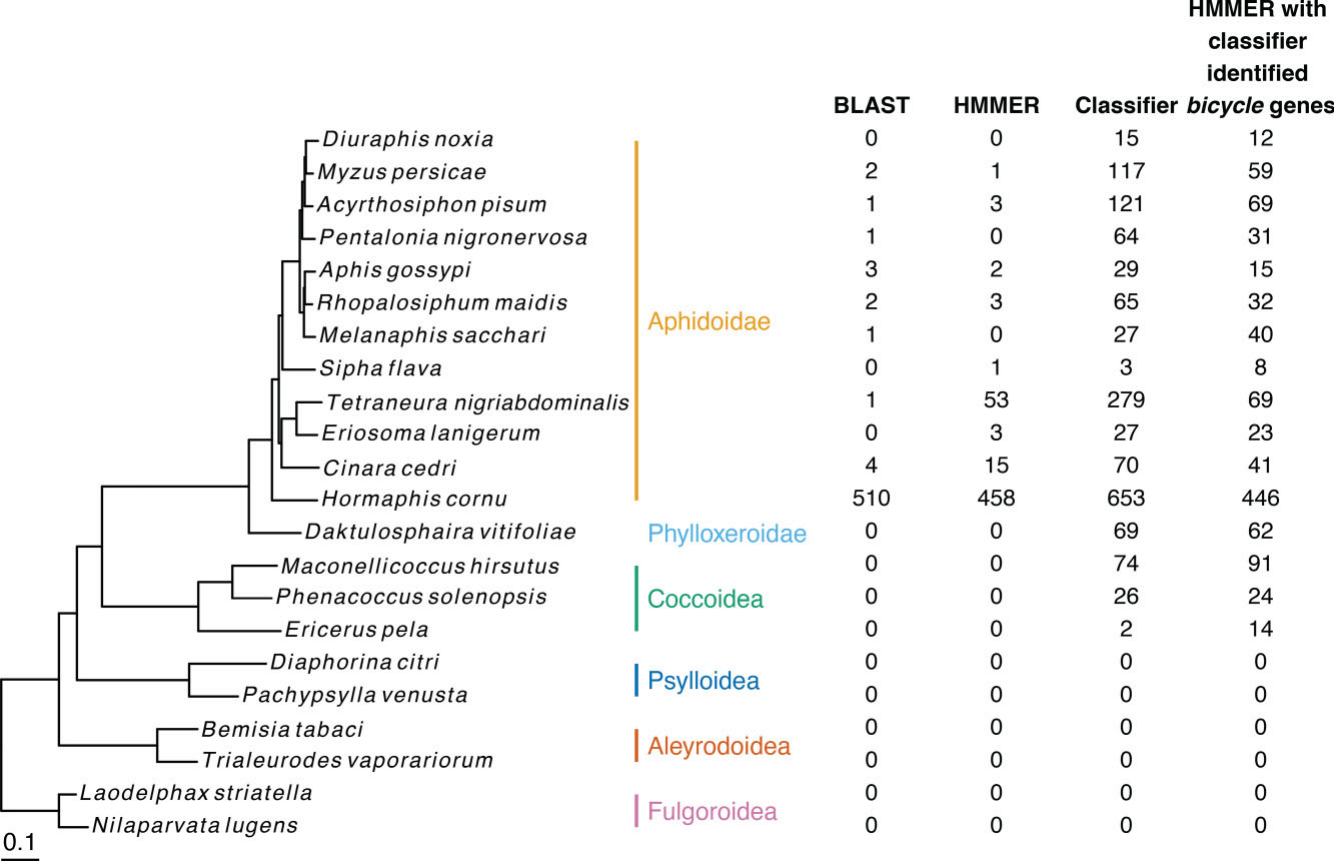
The gene structure-based classifier detects *bicycle* genes in genomes of aphids, phylloxerids, and coccids. A whole-proteome phylogeny of the species studied is shown on the left. Family membership is shown in color beside the species names. The number of candidate *bicycle* homologs detected by *BLAST*, *hmmer*, and the gene structure-based classifier is shown to the right of the phylogeny. A second *hmmer* search was performed starting with the candidate *bicycle* homologs identified by the gene structure-based classifier within each species, and the results of this search are shown in the far right column. Scale bar represents 0.1 substitutions per residue.

*Bicycle* genes in *H. cornu* were identified originally as a subset of previously unannotated genes that were enriched in the salivary gland mRNA of the gall-inducing foundress (Korgaonkar et al., 2021). *Bicycle* genes were found to be the single largest group of such genes that shared sequence similarity. These 476 genes are extremely divergent from one another at the amino-acid sequence level and appear to have evolved rapidly due to positive natural selection. Using sequence-based homology search methods *BLAST* and *hmmer*, we identified 34 additional candidate *bicycle* paralogs in *H. cornu* (using *BLAST*) and some putative homologs in other aphid species (in total 15 with *BLAST* and 81 with *hmmer*). We did not detect any candidate *bicycle* orthologs in genomes from insects of the families Phylloxeridae, Coccoidea, Psylloidea, Aleyrodoidea, and Fulgoroidea (fig. 1).

Gene synteny conservation can be used to identify highly divergent orthologs (Tekaia, 2016). We observed that within the *H. cornu* genome many closely related *bicycle* genes were located on separate chromosomes (supplementary fig. S1A, Supplementary Material online), suggesting that *bicycle* genes undergo transposition at a high rate and that *bicycle* genes are unlikely to display extensive conserved synteny. To more directly test whether conserved gene synteny may allow identification of *bicycle* homologs across the species studied here, we searched for conserved gene synteny near orthologous *bicycle* genes that were identified between *H. cornu* and *Tetraneura nigriabdominalis* (fig. 1) as reciprocal best *phmmer* hits. *Bicycle* orthologs shared many fewer homologous genes in their flanking regions than randomly selected subsets of genes (supplementary fig. S1*B*, Supplementary Material online), further suggesting that conserved synteny is unlikely to be a useful measure to identify *bicycle* homologs across the species studied here. We therefore sought a different method to identify highly divergent *bicycle* homologs.

### A Classifier Based on Only Gene Structural Features Can Identify Bicycle Genes with High Probability

Sequence-based search methods suggested that *bicycle* genes may be found in other species, but the low number of genes identified, and the incongruence between results from *BLAST* and *hmmer* suggested that sequence-based search may provide low sensitivity to detect *bicycle* homologs (Vakirlis et al., 2020; Weisman et al., 2020). We noticed, however, that while sequence divergence was high at the amino-acid level among *H. cornu bicycle* genes, non-sequence specific features of *bicycle* genes appeared to be relatively well conserved. For example, *bicycle* genes contain a large number of unusually small internal exons (Korgaonkar et al., 2021), making them clear outliers to most genes in the *H. cornu* genome. We also observed that almost all internal exons of *bicycle* genes start with codon position 2, that is, they exhibit exon phase 2 (supplementary fig. S1*C*, Supplementary Material online), which is an extremely different distribution of exon phases compared with other genes in the genome (supplementary fig. S1*D*, Supplementary Material online). Genes containing many exons of similar size all with the same phase are rare in most genomes (Ruvinsky and Watson, 2007).

We hypothesized that some aspects of *bicycle* gene structure may evolve more slowly than the primary protein sequences and thus provide an evolutionary signal to detect distantly related *bicycle* homologs (Aguiar et al., 2015). For example, the pattern of exon phases can allow identification of highly divergent homologous genes (Roy and Gilbert, 2005; Ruvinsky and Watson, 2007). We therefore explored whether a linear logistic regression classifier could identify *bicycle* homologs based on the following structural features of each gene: length of gene in genome, from start to stop codons; first and last exon length; internal exon mean length; length of the sum of codons; and number of internal exons that start in phase 0, 1, or 2 (supplementary fig. S2*A*, Supplementary Material online). Since almost all internal *bicycle* exons are of phase 2, there is no additional information from the order of exon phases in *bicycle* genes, and we therefore simply counted the number of exons of each phase.

Characterized *bicycle* genes have an average of approximately 17 and a minimum of 5 exons (supplementary fig. S2*E*, Supplementary Material online), which is strongly different from the genome-wide background (supplementary fig. S2*F*, Supplementary Material online) (two-sample Kolmogorov–Smirnov test *P*-value < 2.2 × 10^−16^), and Bicycle protein lengths are well explained by total exon number (c.f. supplementary fig. S1*G* and S1*H*, Supplementary Material online). We searched predicted genes containing at least four exons so that we could accurately summarize internal exon mean lengths and number of internal exons that start in each of the three phases. Thus, if any *bicycle* genes have evolved to have three or fewer exons, then they could not be detected with this classifier.

The classifier exhibited extremely good performance with all data partitions (supplementary fig. S2*B*, Supplementary Material online) and, therefore, a full model was constructed using all *bicycle* and annotated genes. (Non-annotated genes were excluded from model training and validation because we hypothesized that this set may include additional *bicycle* homologs.) The classifier categorized the vast majority of genes as either non-*bicycle* or *bicycle* genes with high probability (supplementary fig. S2*C*, Supplementary Material online). To decide on a probability cutoff to identify putative *bicycle* homologs, we considered the trade-off between precision (the proportion of true positives among both true and false positives) and recall (the proportion of true positives among true positives and false negatives). We chose a model probability cutoff that equalized precision and recall (P/R = 1), which yielded precision and recall values of 0.99 (supplementary fig. S2*D,E*, Supplementary Material online).

To determine whether all gene features were required for accurate classification, we removed one factor at a time, retrained the model, and calculated precision and recall values using the same threshold as the full model. We found that no single factor was solely necessary for model performance (supplementary fig. S2*F*, Supplementary Material online), although removal of the number of mode 2 exons resulted in the strongest decrement in model performance, resulting in precision and recall values of 0.98 and 0.96, respectively.

To determine whether any single predictor was sufficient to identify *bicycle* genes with high confidence, we calculated precision and recall values for linear models using one predictor at a time (supplementary fig. S2*G*, Supplementary Material online). Mean internal exon length alone performed best (supplementary fig. S2*G*, Supplementary Material online), consistent with the observation that *bicycle* genes contain an unusually large number of small exons (supplementary fig. S3, Supplementary Material online). The number of internal exons in mode 2 also displayed some predictive power on its own (supplementary fig. S2*G*, Supplementary Material online). No other predictors alone had substantial predictive power for discriminating *bicycle* genes from the background (supplementary fig. S2*G*, Supplementary Material online). The observation that no single predictor was solely necessary for performance but two predictors were individually sufficient to provide good predictive power indicates some correlation between the predictor variables (supplementary fig. S2*H–J*, Supplementary Material online). Since no predictors were scalar multiples of each other, we employed the full model using all predictors to search for new *bicycle* genes.

### Additional Candidate Bicycle Genes Can be Found in Many Other Species

To search for candidate *bicycle* genes in other species, we applied the classifier to all predicted genes from the genomes of twenty-two species spanning the Aphidoidea and species from the five most closely related families, the Phylloxeroidea, Coccoidea, Psylloidea, Aleyrodoidea, and Fulgoroidea (fig. 1). Multiple candidate *bicycle* genes were identified in all Aphidoidea, Phylloxeroidea, and Coccoidea we studied and the candidate *bicycle* genes identified by the classifier encompass 98.2–100% of the *BLAST* identified *bicycle* genes and 86.8-100% of the *HMMER* identified *bicycle* genes in all species with more than three *bicycle* genes. This concordance of methods supports the inference that 1) the classifier has identified *bicycle* homologs and 2) the classifier has higher power than sequence-based approaches to identify *bicycle* genes. As expected from the features of the classifier, the candidate *bicycle* genes in all species exhibited the unusual feature of being encoded by genes containing many micro-exons (supplementary fig. S3, Supplementary Material online).

While the classifier exhibits substantial power to detect highly divergent *bicycle* genes across species, the classifier might fail to identify *bicycle* genes with gene structures that are divergent from the *bicycle* genes originally identified in *H. cornu* that were used to train the classifier. In addition, the genomes of many species studied here are highly fragmented, and some true *bicycle* genes may have been annotated with a subset of their exons. We therefore performed a second profile *hmmer* search within each species starting with the classifier-identified *bicycle* genes in each species (last column in fig. 1). This second *hmmer* search identified additional candidate *bicycle* genes in some species, suggesting that the classifier underestimates the number of *bicycle* genes in some species.

### Candidate Bicycle Genes in *H. cornu* are Highly Expressed and Share Structural Homology

When we applied the classifier to all genes in the *H. cornu* genome, we identified 653 candidate *bicycle* genes, compared with the 476 *bicycle* genes we described in our original study (Korgaonkar et al., 2021). Four originally defined *bicycle* genes were not detected and are thus false negatives. In addition, 181 newly detected *bicycle* genes were detected and are thus “false positives.” Since we chose a probability cutoff that should report an approximately equal number of false negatives and false positives, we hypothesized that the vast majority (approximately 181 - 4) of “false positives” are newly discovered *bicycle* homologs.

**Fig. 2.**
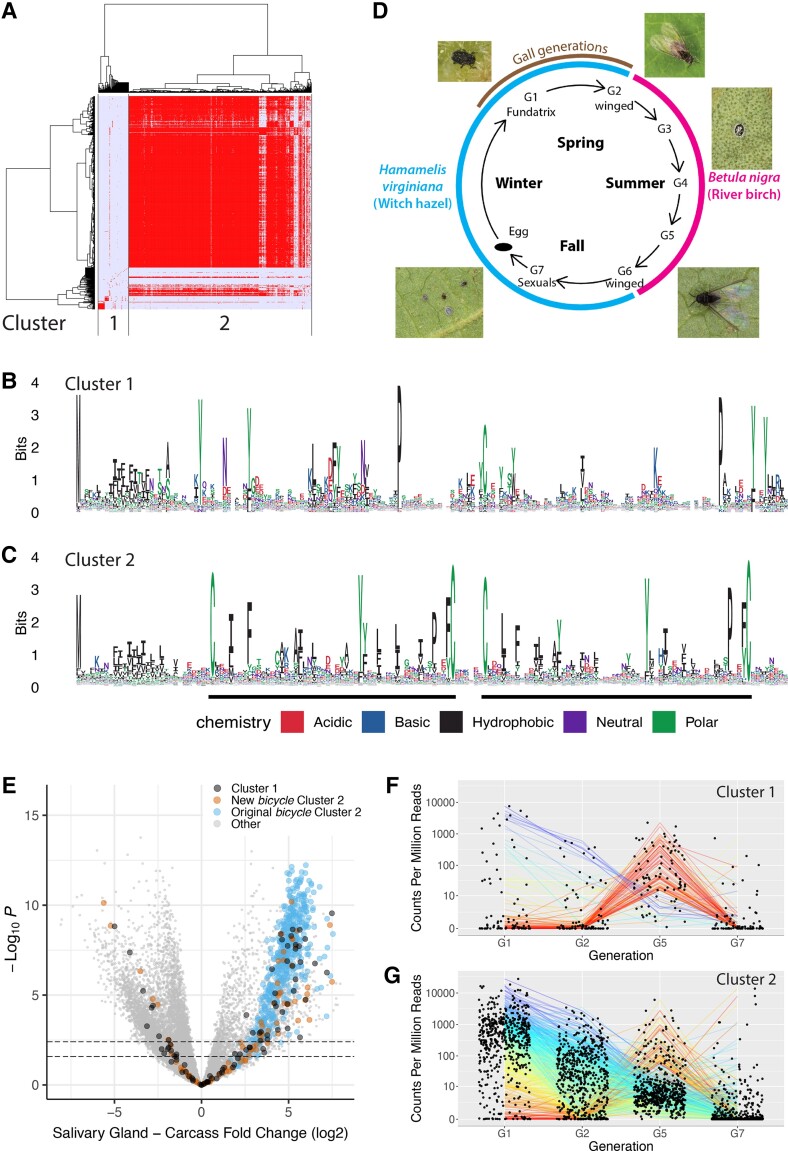
The gene-structure based classifier identifies additional CYC and non-CYC motif containing genes in the *H. cornu* genome that may contribute to the effector protein repertoire of multiple stages of the *H. cornu* life cycle. (*A*) Hierarchical clustering of candidate *H. cornu* bicycle homologs detected by the gene-structure based on amino-acid sequence similarity measure reveals two clusters of genes with similar sequences. Some of the genes in Cluster 1 appear to have some sequence similarity to Cluster 2 genes. (*B*) Genes in Cluster 1 encode proteins with N-terminal signal sequences and weak similarity to *bicycle* genes. (*C*) Genes in Cluster 2 encode proteins with sequence similarity to the previously described *bicycle* genes. (*D*) Diagram of life cycle of *H. cornu*. Individuals from different generations exhibit phenotypes that are specialized for each stage of the complex life cycle. The fundatrix (G1) is the only generation that induces a gall. This panel was published previously as Figure 1J in Korgaonkar et al. (2001) and is available under Creative Commons Attribution License (CC BY 4.0). (*E*) A volcano plot of the strength of evidence for differential expression (-log10(P value)) versus the fold-change differential expression of salivary glands isolated from fundatrices versus sexuals reveals that some of the Cluster1 (black) and 2 (orange) genes identified by the gene structure based classifier are as strongly enriched in fundatrix salivary glands as the originally described *bicycle* genes (blue). In addition, several Cluster 1 and 2 genes are more strongly enriched in salivary glands of sexuals. (*F–G*) Plots of gene expression levels for Cluster 1 (*F*) and 2 (*G*) genes across four generations of the life cycle reveals that while most *bicycle* homologs are most strongly expressed in fundatrix salivary glands (e.g. purple lines), some are weakly expressed in fundatrix salivary glands and then strongly expressed in salivary glands of individuals from other stages of the life cycle (e.g. red lines).

We clustered these newly discovered *bicycle* genes together with the original *bicycle* genes based on sequence similarity (fig. 2*A*) and found that 563 represented a single group of related genes (Cluster 2, fig. 2*A*) that all share sequence similarity with, and included, all but one of the originally defined 476 *bicycle* genes (fig. 2*C*).

We also identified a second cluster of 94 genes that encode proteins that do not share strong sequence similarity with the originally defined Bicycle proteins and contain degenerated CYC motifs (Cluster 1, fig. 2*A* and 2*B*). Approximately 20 of these genes were strongly enriched in the salivary glands of gall-inducing foundresses, but many of these Cluster 1 genes were strongly enriched in salivary glands of other life stages, especially generations that feed on *Betula nigra* (River Birch) (fig. 2*D–F*). In addition, approximately 20 *bicycle* genes containing the CYC motif (Cluster 2) were weakly expressed in fundatrix salivary gland, but strongly expressed at other life stages (fig. 2*E–G*).

Like the original 476 *bicycle* genes, the new putative *bicycle* homologs likely experienced increased levels of positive selection. We found that the new putative *bicycle* homologs displayed a similar pattern to the original *bicycle* genes of elevated ratios of non-synonymous (*d_N_*) to synonymous (*d_S_*) substitutions between *H. cornu* and its closely related sister species *H. hamamelidis* compared to the genomic background (supplementary fig. S4*A–C*, Supplementary Material online). In addition, we performed a genome-wide selection scan using a site frequency spectrum-based composite likelihood ratio test that calculates the likelihood ratio of a sweep at a certain location in the genome to the neutral model. We found that signals of selective sweeps are enriched near both the original *bicycle* genes and the newly discovered putative *bicycle* homologs (supplementary fig. S4*D*, Supplementary Material online).

Since some of these new putative *bicycle* homologs shared little apparent sequence similarity with the originally defined *bicycle* genes, we sought additional evidence for homology. We hypothesized that other details of gene structure not employed in the classifier, such as intron positions, might provide additional evidence of shared ancestry. We therefore performed gene-structure-aware multiple sequence alignment of the original *bicycle* genes and the new putative *bicycle* genes identified by the classifier (Gotoh, 2021).

**Fig. 3.**
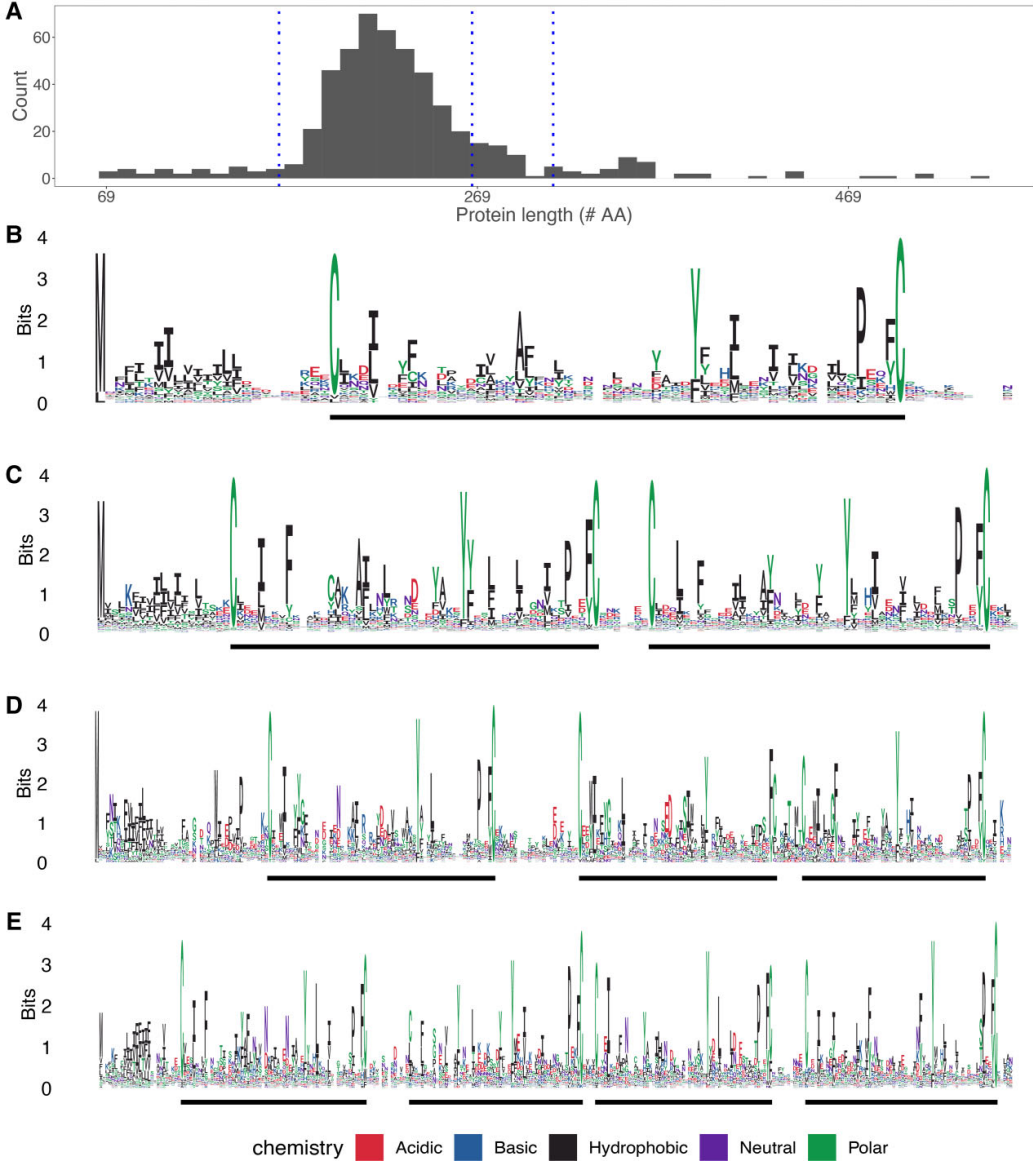
*H. cornu bicycle* genes belong to four major categories, *unicycle, bicycle, tricycle,* and *tetracycle* genes. (*A*) The originally identified *bicycle* genes encode proteins that exhibit a wide size range, with representatives of approximately four size classes, indicated by the vertical dotted blue lines. (*B–E*) Logo plots of the proteins from each of the four size classes of Bicycle proteins identifies unicycle (*B*), bicycle (*C*), tricycle (*D*), and tetracycle (*E*) proteins. The CYC motifs in each protein are identified by thick black lines below each Logo plot.

Initial attempts to align all 657 candidate proteins were uninformative, and we hypothesized that this was because these proteins display a large range of lengths and exon numbers (supplementary fig. S4*E*, Supplementary Material online). We therefore divided Bicycle proteins of Cluster 2 into four groups based on protein length (blue dotted lines in fig. 3*A*). Logo plots of these four groups revealed a striking pattern, proteins contain one, two, three, or four CYC motifs (fig. 3*B–E*), and we therefore call these *unicycle, bicycle, tricycle,* and *tetracycle* genes, respectively. This evolutionary comparison suggests that the CYC motif is a functional unit, which can be multimerized within individual proteins.

We then divided *H. cornu bicycle* homologs into four length categories and performed gene-structure aware protein sequence alignments to search for shared intron position between the originally defined *bicycle* genes and the newly discovered *bicycle* putative homologs of Cluster1. We identified many evolutionarily shared intron positions between the two classes of putative homologs (supplementary fig. S5, Supplementary Material online).

To determine whether more introns are shared between the original *bicycle* genes and newly identified putative *bicycle* genes than expected by chance—for example given an alignment of two groups of unrelated genes—we developed a metric of intron concordance and estimated the null distribution by resampling intron concordance between *bicycle* genes and three unrelated gene families (supplementary fig. S6, Supplementary Material online). Intron concordance was measured as the correlation coefficient between the number of genes sharing an intron at each alignment position for one gene family versus the *bicycle* gene family (supplementary fig. S6*A–D*, Supplementary Material online). Unrelated gene families were found to have intron positions distributed approximately randomly relative to *bicycle* genes with mean R close to 0 (supplementary fig. S6*C,E*, Supplementary Material online). In contrast, newly discovered *bicycle* genes, both from *H. cornu* and from other species, displayed positive R much greater than 0 and significantly different from distributions of R values found for pairs of unrelated gene families (supplementary fig. S6*E*, Supplementary Material online). This analysis provides statistically significant support for the conclusion that newly discovered putative *bicycle* genes share introns in the same locations more often than expected by chance between unrelated gene families and thus that they are likely *bicycle* gene homologs.

These results provide evidence for several conclusions. First, measures of gene structure alone can reliably identify many, but perhaps not all, divergent *bicycle* genes. Second, some *bicycle* homologs have evolved highly divergent sequences, raising the possibility that highly divergent homologs may be present in other species but are undetectable with sequence-similarity search algorithms. Third, some *bicycle* homologs are highly expressed in salivary glands of non-gall forming life stages, suggesting that some of these proteins may contribute to the effector-protein repertoire outside of the context of gall development. Given these observations, we next tested whether the classifier could identify *bicycle* homologs in a divergent galling aphid species.

### Many Candidate Gall Effector Genes in Tetraneura nigriabdominalis Are Highly Divergent Bicycle Genes

There are two major clades of gall forming aphids, the Hormaphididae, to which *H. cornu* belongs, and the Pemphigidae. These two aphid families are thought to have shared a common ancestor that induced galls. Therefore, to determine whether *bicycle* genes were present in the common ancestor of gall forming aphids, we assembled and annotated the genome of *T. nigriabdominalis* (supplementary fig. S6*A*, Supplementary Material online), a gall forming aphid belonging to the Pemphigidae (Álvarez et al., 2013). Details of the genome assembly can be found in Supplementary Methods, Supplementary Material online. We annotated the *T. nigriabdominalis* genome using mRNA sequencing reads generated from salivary glands and carcasses of the fundatrix (G1) and G2 generations (supplementary fig. S6*B*, Supplementary Material online).

**Fig. 4.**
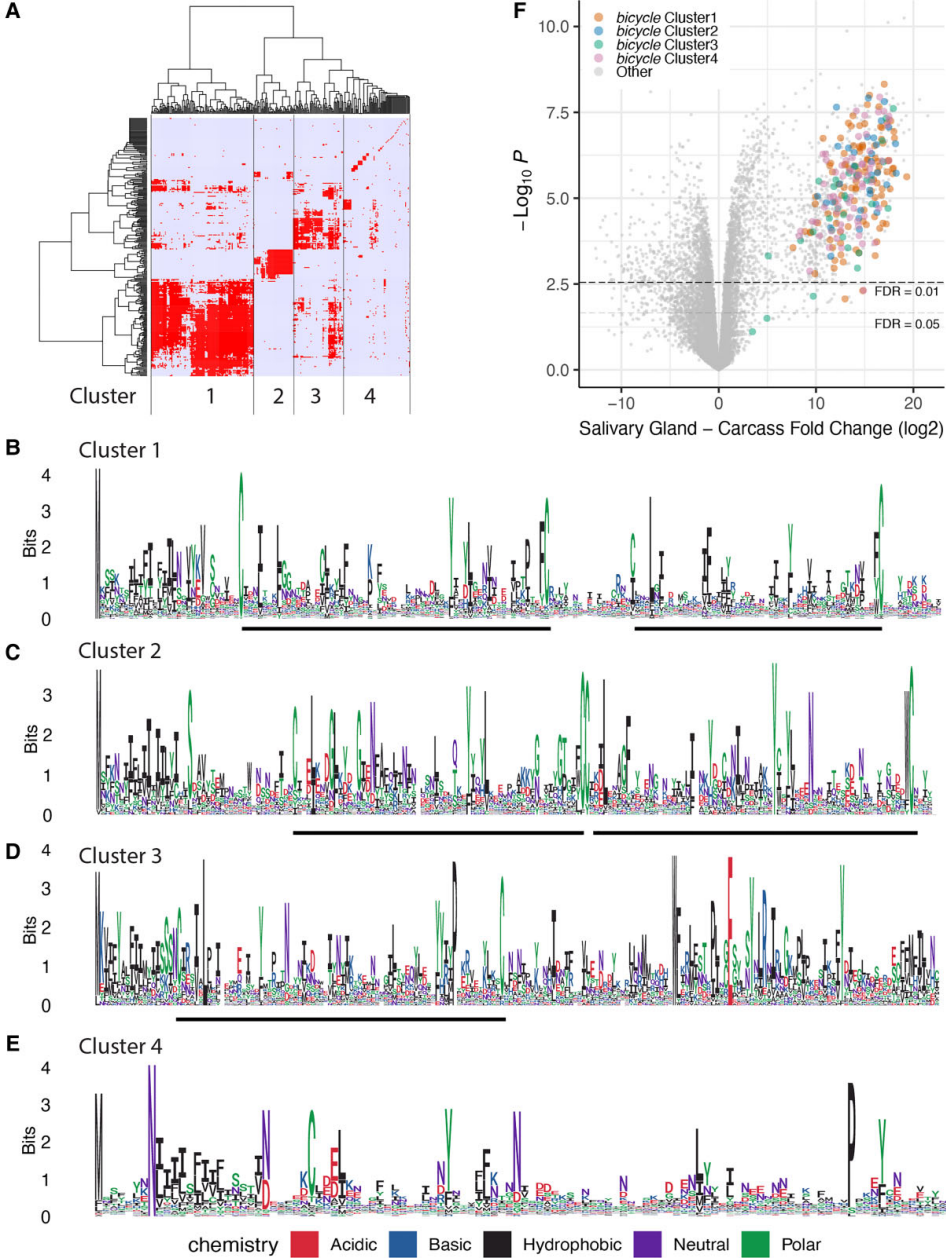
The *T. nigriabdominalis bicycle* homologs detected by the gene structure-based classifier display CYC motifs and highly divergent protein sequences. (*A*) Hierarchical clustering of candidate *T. nigriabdominalis bicycle* homologs based on amino-acid sequence similarity measure reveals four clusters of genes. (*B–E*) The four clusters of *T. nigriabdominalis bicycle* homologs display two (*B*,*C*) or one (*D*) CYC motif, or no clear CYC motifs (*E*). The cluster identities are indicated in each subplot. (*F*) Volcano plot of genes differentially expressed in fundatrix salivary glands versus carcass with *bicycle* genes labelled. Most of the *T. nigriabdominalis* candidate *bicycle* homologs are strongly over-expressed in the fundatrix salivary glands.

We applied the *H. cornu bicycle* gene classifier to predicted genes of the *T. nigriabdominalis* genome and identified 279 candidate *bicycle* homologs (fig. 1). In contrast, sequence-based search using BLAST and *hmmer* identified 1 and 53 candidate homologs at *E*-value < 0.01, respectively. We clustered the 279 *T. nigriabdominalis* genes discovered by the gene-structure based classifier based on their predicted amino acid sequences (fig. 4*A*) and identified three clusters that included proteins with clear evidence for CYC motifs (fig. 4*B–D*). One cluster of 94 genes does not exhibit clear CYC motifs (fig. 4*E*). The extreme sequence divergence of these genes within *T. nigriabdominalis* is supported by the fact that profile *hmmer* search recovered only 69 of these original 279 genes (fig. 1). To test whether these genes likely shared a common ancestor, we performed gene-structure aware alignments of proteins with and without CYC motifs and identified more shared introns between putative homologs with and without CYC motifs (supplementary fig. S6*C*, Supplementary Material online) than expected by chance (supplementary fig. S6*E*, Supplementary Material online). Thus, the *T. nigriabdominalis* genome contains apparent *bicycle* genes that have become essentially unrecognizable as *bicycle* homologs at the sequence level.

To explore whether the *T. nigriabdominalis bicycle* genes, and especially the divergent putative *bicycle* homologs, might act as effector proteins, we dissected salivary glands from fundatrices and compared mRNA expression levels in salivary glands versus carcasses. Almost all of the *bicycle* homologs detected by the classifier are amongst the most strongly over-expressed genes in the fundatrix salivary glands (fig. 4*F*), suggesting that they may contribute to the effector protein repertoire in *T. nigriabdominalis*.

The discovery of *bicycle* genes in *T. nigriabdominalis* using the gene-structure based classifier provides evidence that the classifier can identify putative *bicycle* homologs even when the genes cannot be identified using sequence-based homology methods and that *bicycle* genes were likely present in the common ancestor of gall-forming aphids. We therefore next tested whether the classifier can detect putative *bicycle* homologs in an aphid species that does not induce galls.

### The Pea Aphid (*Acyrthosiphon pisum*) Genome Includes Many Bicycle Homologs That Are Candidate Effector Proteins

**Fig. 5.**
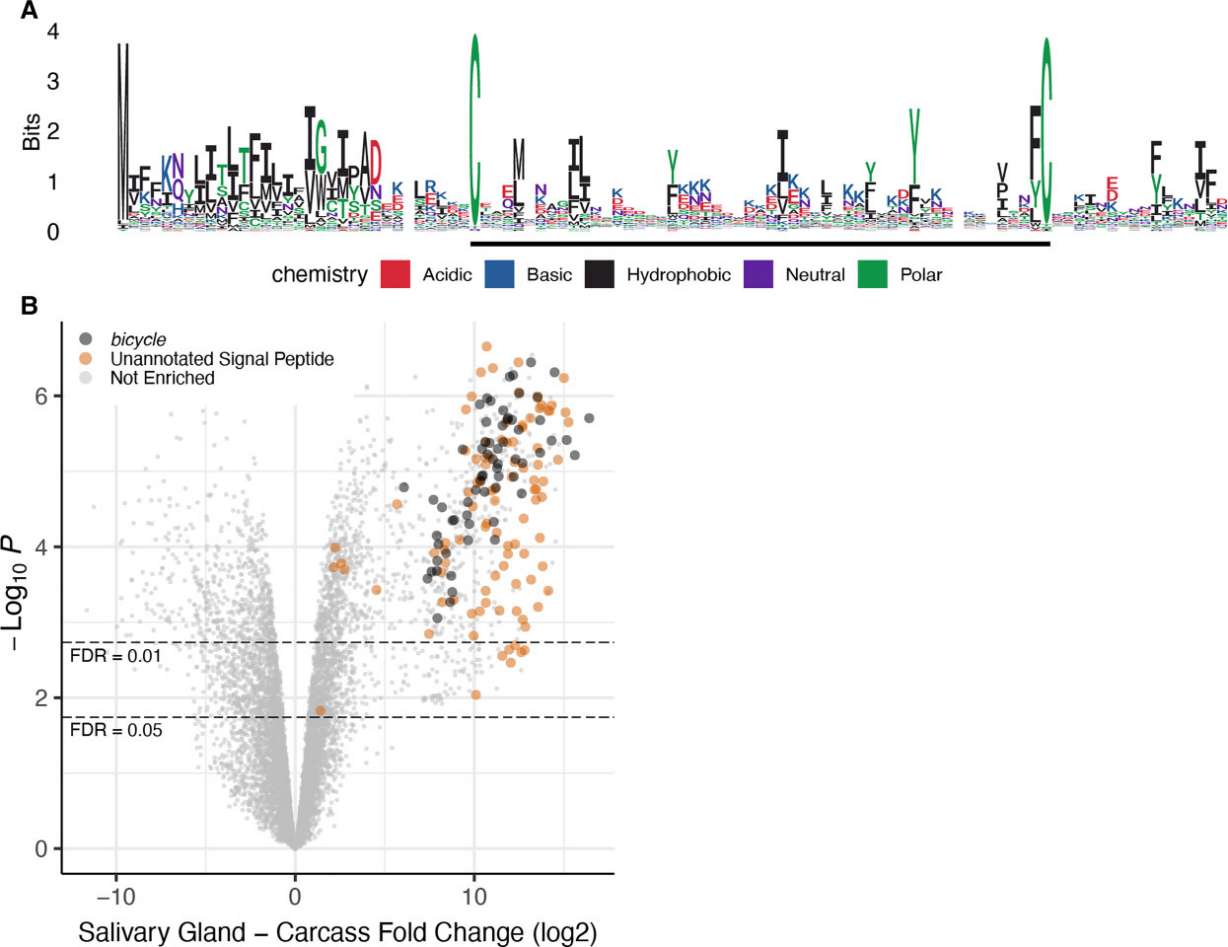
*A. pisum bicycle* homologs contain a single CYC motif and are strongly enriched in salivary glands. (*A*) Logo plot of *A. pisum bicycle* homologs detected by gene structure-based classifier. Most *A. pisum* homologs contain a single CYC motif. (*B*) Volcano plot of differential expression between salivary glands and carcass reveals that *A. pisum bicycle* homologs are strongly over-expressed in salivary glands.

The gene-structure based classifier detected 121 putative *bicycle* homologs in the genome of *A. pisum*, a species that does not induce galls (Richards et al., 2010; Li et al., 2019). In contrast, sequence-based search using *BLAST* and *hmmer* identified 1 and 3 candidate hits at *E*-value < 0.01, respectively. Multiple sequence alignment of the 121 *A. pisum* putative *bicycle* homologs revealed that most of these genes encode proteins with N-terminal secretion signal sequences and a single CYC motif (fig. 5*A*). Gene structure aware alignment identified more shared introns between *H. cornu bicycle* genes and *A. pisum* putative *bicycle* homologs (supplementary fig. S8, Supplementary Material online) than expected by chance (supplementary fig. S6*E*, Supplementary Material online), providing independent confirmation that these genes shared a common ancestor.

To explore whether these *A. pisum bicycle* homologs may contribute to the effector gene repertoire of *A. pisum*, we performed RNA-seq of salivary glands and carcasses. We found that 55% of the putative *bicycle* genes are among the most strongly overexpressed genes in salivary glands, along with a similar number of additional unannotated genes with signal peptides (fig. 5*B*). In addition, 17 proteins encoded by genes with multiple microexons were identified by a previously-published proteomic study of proteins secreted by *A. pisum* into an artificial food medium (Dommel et al., 2020), and we found that at least 13 of these proteins are *bicycle* homologs (supplementary table S1, Supplementary Material online). In summary, the *A. pisum* genome encodes approximately 121 *bicycle* homologs that may contribute to the *A. pisum* effector protein repertoire.

### Bicycle Genes Are Present in Genomes of Aphids, Phylloxerids, and Coccids

The presence of *bicycle* homologs in three divergent aphid species, *H. cornu*, *T. nigriabdominalis*, and *A. pisum* implies that *bicycle* genes were present in the common ancestor of aphids, which lived approximately 280 MYA. To further explore the origins and evolution of *bicycle* genes, we downloaded genomes and RNA-seq data for nine additional aphid species and ten outgroup species from NCBI. We annotated predicted genes in all genomes using the same bioinformatic pipeline that we have used previously to discover *bicycle* genes in other species, applied the *bicycle* gene classifier to all predicted genes, and manually annotated all putative *bicycle* homologs guided by RNA-seq data. One caveat of this analysis is that many of these genomes are fragmented into many contigs and genes bridging contigs are often misannotated or not annotated, resulting in possible under-counting of *bicycle* homologs. In addition, we detected multiple putative *bicycle* homologs near the ends of contigs, suggesting that parts of single *bicycle* homologs are present on multiple contigs, which would lead to over-counting *bicycle* gene homologs. While these problems with current annotations are expected to increase variance of estimates of the number of *bicycle* homologs, we did not detect a dependence of number of *bicycle* homologs detected on genome assembly quality (supplementary fig. S9*A*, Supplementary Material online) or on the number of genes annotated for each genome (supplementary fig. S9*B*, Supplementary Material online). In addition, we employed chromosome-level genome assemblies for two outgroup species where no predicted *bicycle* genes were found, *P. venusta* (Li et al., 2020) and *T. vaporariorum* (Xie et al., 2020), suggesting that the failure to identify *bicycle* genes in these species did not result from poor genome assemblies.

We estimated the phylogeny for these 22 species using whole-genome proteomic predictions (Emms, 2019), and this phylogeny is in general agreement with earlier studies based on a small number of genes (von Dohlen and Moran, 1995; Nováková et al., 2013; Johnson et al., 2018) (fig. 1). We detected putative *bicycle* homologs in all aphid species studied here, supporting the inference that *bicycle* genes were present in the common ancestor of aphids (fig. 1). We found extensive variation in the number of *bicycle* genes between species. In all aphid species with a sufficient number of *bicycle* homologs, multiple sequence alignment revealed the presence of CYC domains (supplementary fig. S9*C–F*, Supplementary Material online). In most Aphidini species (e.g., *M. persicae*, *A. pisum*, *P. nigronervosa*), most homologs appear to be *unicycle* genes (supplementary fig. S9*C,D*, Supplementary Material online). In *Cinara cedri*, many homologs appear to be *tetracycles* (supplementary fig. S9*F*, Supplementary Material online).

We found 69 putative *bicycle* homologs in *Daktulosphaira vitifoliae* indicating that *bicycle* genes were present in the common ancestor of the Aphidomorpha (Aphidoidea + Phylloxeridea). We also found 74, 26 and 2 putative *bicycle* homologs in the coccid species *Maconellicoccus hirsutus*, *Phenacoccus solenopsis*, and *Ericerus pela*, respectively. In *M. hirsutus* and *E. pela* profile *hmmer* search with the classifier identified genes identified 17 and 12 additional candidate *bicycle* genes, respectively. The putative *bicycle* homologs from these non-aphid species do not contain obvious CYC motifs (supplementary fig. S9*A–C*, Supplementary Material online). However, gene structure aware alignment with the *H. cornu bicycle* genes revealed that the putative homologs from *D. vitifoliae*, *M. hirsutus*, and *P. solenopis* share more introns in the same locations with *H. cornu bicycle* genes (supplementary fig. S10*D–F*, Supplementary Material online) than expected by chance (supplementary fig. S10*E*, Supplementary Material online), supporting the inference that these are *bicycle* homologs.

We did not detect *bicycle* homologs in any of the species sampled from the Psylloidea, Aleyrodoidea, or Fulgoroidea. These results indicate that *bicycle* genes were present in the common ancestor of coccids and aphids and may have evolved in the common ancestor of these lineages. However, for at least two reasons we cannot rule out the possibility that *bicycle* genes originated earlier. First, we observed extensive variation in the number of *bicycle* homologs across lineages, so *bicycle* genes may have been lost in the specific outgroup species studied here. Second, ancestral *bicycle* genes may not display the specific gene structure detected by our classifier. Deeper taxonomic sampling and other homology search approaches may reveal an older origin of *bicycle* genes.

### Aphid Genomes Contain a Conserved *Megacycle* Gene

**Fig. 6.**
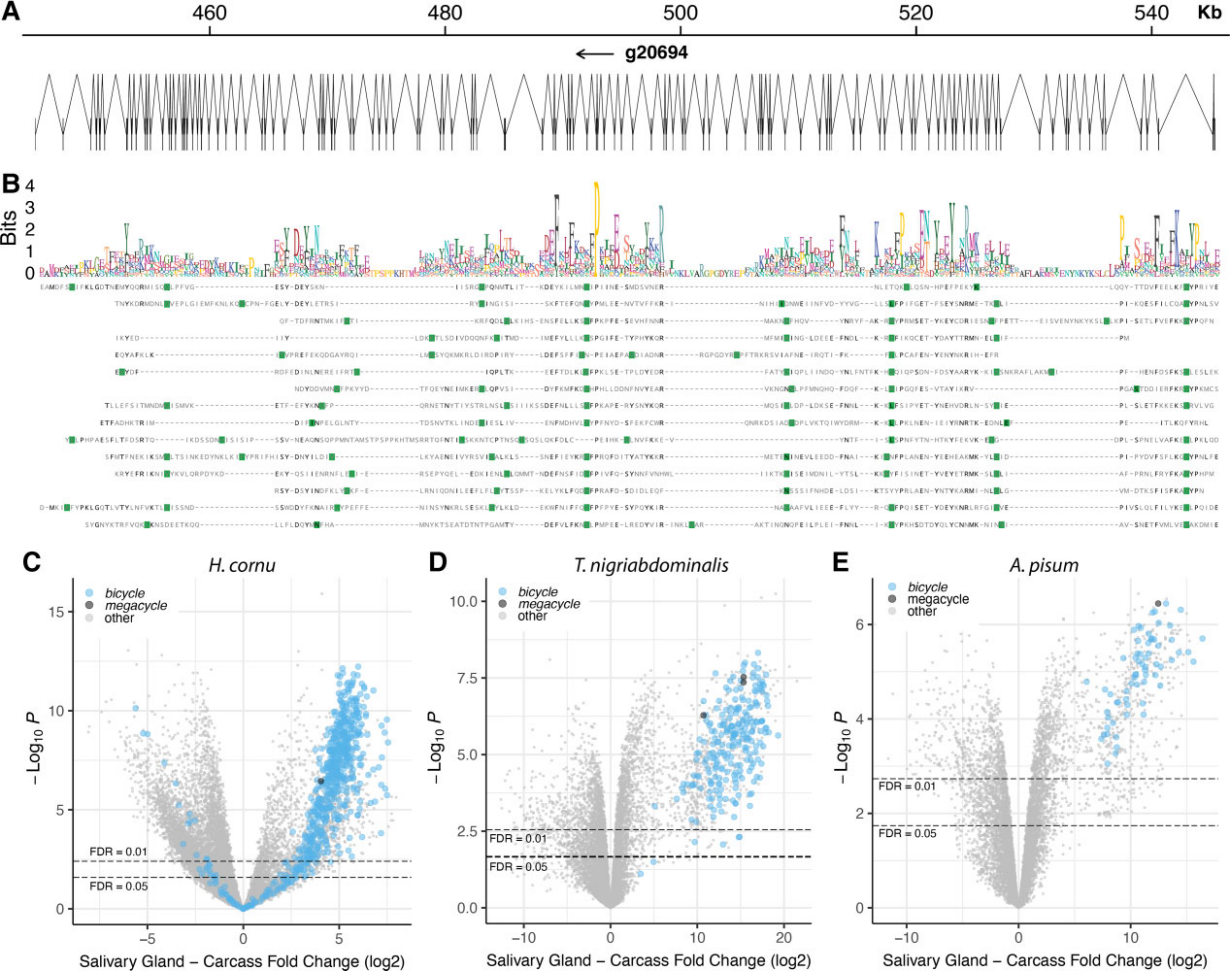
*Megacycle* genes have a repeating protein structure and are overexpressed in salivary glands of multiple aphid species. (*A*) Gene structure of the *M. sacchari megacycle* gene (*g20694*). The gene includes 118 exons encoded in approximately 100 kbp of DNA. The protein is 2092 AA long. (*B*) Rapid automatic detection and alignment of repeats (RADAR) analysis of the *M. sacchari megacycle* gene provides evidence for 15 repeating units within the protein encoded by *g20694*. Multiple sequence alignment of these repeat units (not using information on gene structure) reveals that many introns, marked in green, are located in identical or similar positions in the aligned sub-sequences. A logo plot of the aligned repeated sequences is shown above the alignment. There is no obvious evidence for CYC motifs, although the motif is approximately the same length as the proteins encoded by many *bicycle* genes. (*C–E*) Volcano plots of differential expression between salivary glands and carcasses for three species, *H. cornu* (*C*), *T. nigriabdominalis* (*D*), and *A. pisum* (*E*), reveals that *megacycle* genes (black dots) are strongly over-expressed in salivary glands of all three species. *Bicycle* genes are marked as light blue dots.

In 11 of the 12 aphid genomes we studied, our classifier identified a single extremely large gene containing more than 100 microexons and encoding a protein of approximately 2000 amino acids as a candidate *bicycle* homolog (fig. 6*A*). Since this gene was so large and most existing aphid genomes are incompletely assembled, we observed that many of these gene models were incomplete. We therefore performed *de novo* transcript assembly for all species studied using Trinity (Grabherr et al., 2011) and manually assembled consensus transcripts of these long candidate *bicycle* genes.

Since we had previously found that *bicycle* gene length is correlated with the number of encoded CYC motifs (fig. 3), we explored whether these genes encoded proteins with multiple repeating units. Using Rapid Automatic Detection and Alignment of Repeats (Heger and Holm, 2000) via EMBL's European Bioinformatics Institute analysis tool (EMBL-EBI) (Madeira et al., 2019), we found that these large proteins contain an N-terminal secretion signal and many repeating motifs and each motif is approximately the size of two CYC motifs. This structure was most obvious for the *Melanaphis sacchari* gene (fig. 6*A*). Sequence-based alignment of 15 predicted repeats of the *M. sacchari* protein, which did not use information of intron positions, revealed multiple shared intron locations across repeats (fig. 6*B*). Thus, this protein appears to have evolved through multiple internal tandem duplications. This large protein exhibited little obvious sequence similarity to *bicycle* genes, but gene-structure aware alignment revealed multiple apparently conserved intron positions shared between the internal duplications of the large gene from *M. sacchari* and *H. cornu bicycle* genes (supplementary fig. S11*A*, Supplementary Material online), suggesting that they share a common ancestor.

These large genes appear to be divergent Bicycle proteins that have duplicated the core motif many times. We therefore call these *megacycle* genes. Compared to most *bicycle* genes, *megacycle* orthologs are relatively well conserved across aphids and a phylogenetic tree reconstructed from the inferred Megacycle proteins is similar to the aphid phylogeny estimated from the whole proteome (supplementary fig. S11*B*, Supplementary Material online). Most aphid genomes we examined contain a single copy of this gene; we did not detect this gene in *Diuraphis noxia*, and we found three paralogs in both *C. cedri* and *T. nigriabdominalis*. No *megacycle* orthologs were found in any of the non-aphid species.

For the three species for which we have good salivary gland transcriptomic data, *H. cornu*, *T. nigriabdominalis,* and *A. pisum*, the *megacycle* genes are strongly expressed in salivary glands (fig. 6*C–E*). Thus, *megacycle* genes may contribute to the effector protein repertoire in aphids. The relatively conserved lengths and sequences of *megacycle* homologs suggests that they may share a relatively conserved role across aphids.

## Discussion

Some gene features, such as the structure of the encoded protein or the intron-exon structure of the gene, can provide evidence for homology that is independent of sequence (Bazan, 1991; Brown et al., 1995; Betts, 2001). This source of information is becoming increasingly available with the recent availability of many well-assembled, annotated genomes. It is possible to conceptualize a model that jointly utilizes both sequence and exon-intron structure in homology search, but we realized that *bicycle* genes are encoded with extremely unusual intron-exon structures and that a model incorporating only general features of exons may allow detection of distant homologs.

We found that a linear classifier using information on gene length, exon sizes, exon numbers, and exon phases provided a highly accurate predictor of *bicycle* homologs that could not be identified using sequence similarity searches. This predictor allowed identification of *bicycle* homologs across aphids and in several outgroup taxa (fig. 1). Homology was supported by *post hoc* observation of CYC motifs and of an excess of shared introns across many homologs. Thus, sequence independent features provide substantial evidence of homology and integration of even a few sequence independent features into existing sequence-based search methods may substantially increase power to detect homology relationships between genes. All gene structure features that we used contributed to classifier performance, but no single feature was critically important for classifier performance. Therefore, in future work it would be ideal to incorporate multiple features of gene structure into homology search models.

For several reasons, our classifier may have underestimated the number of *bicycle* homologs in each species. First, this approach is sensitive to the quality of genome annotation, which is itself dependent on genome assembly quality. We found that most automatically annotated gene models of *bicycle* genes required manual re-annotation. Thus, we may have overlooked additional *bicycle* genes in these genomes because of genome fragmentation and inaccurate automated gene annotation. Second, *bicycle* gene annotation in most species is likely hampered by the lack of salivary gland RNA-seq samples. We observed considerable variation in *bicycle* gene expression levels among samples, and deep sequencing of diverse samples is likely required to provide sufficient RNAseq evidence to build accurate *bicycle* gene models. Finally, it is possible that some *bicycle* homologs have evolved both divergent sequences and novel gene structures, preventing identification with any existing method.

### Bicycle Genes are Not Strictly Associated with Gall Formation

*Bicycle* genes were detected originally as putatively secreted proteins strongly expressed in the salivary glands of the gall-forming generation of the aphid *H. cornu* and variation in one *bicycle* gene is genetically associated with gall color (Korgaonkar et al., 2021). These observations led to the hypothesis that many *bicycle* genes participate in gall development. Our observations provide a revised assessment of potential Bicycle protein functions.

First, we found that many *bicycle* genes are most strongly expressed in salivary glands of generations of *H. cornu* that do not induce galls (fig. 2*F–G*). Second, two of the aphids studied here, *H. cornu* and *T. nigriabdominalis*, induce complex galls and their genomes include many more *bicycle* genes than other species we examined, but the genomes of two other gall-forming species, *E. lanigerum* and *D. vitifoliae* have only 27 and 69 *bicycle* genes, respectively. In addition, the genomes of some non-galling aphids have many *bicycle* genes, such as *Myzus persicae* and *A. pisum*, with 117 and 121, respectively. Thus, across species there does not appear to be a strong correlation between the number of *bicycle* genes and the gall-forming habit.

Given these new observations, we hypothesize that if Bicycle proteins have conserved molecular functions, then they likely have different targets in different plant species. In some cases, the targets may induce patterned cell proliferation, resulting in galls, and in others the targets may alter plant physiology to confer benefits on aphids.

None of the three coccid species we studied here induce galls. However, many coccids induce depressions, swellings, or other changes in plant organs (Beardsley and Gonzalez, 1975), including the induction of elaborate galls (Cook and Gullan, 2004). Parr (1940) reported a remarkable series of experiments that appear to demonstrate that extracts of salivary glands of a coccid are sufficient to induce pit galls on oak trees. It may be worth exploring the hypothesis that *bicycle* genes contribute to the effector gene repertoire of scale insects and especially of the gall-forming species.

### New Experimental Opportunities Provided by the Presence of Bicycle Genes in Many Species

One of the challenges with studying the function of *bicycle* genes is that initially they were thought to be restricted to the non-model gall-forming insect *H. cornu*. *H. cornu* displays a complex life cycle, exhibiting many polyphenic forms and migration between two trees every year (Korgaonkar et al., 2021), and prospects for laboratory rearing and subsequent experimental manipulations of this species were poor.

The discovery of *bicycle* genes in other insects opens up the possibility that the function of *bicycle* genes can be studied in species more amenable to laboratory manipulation. For example, RNAi allows gene knockdown in *M. persicae* (Pitino et al., 2011; Coleman et al., 2015; Mathers et al., 2017; Niu et al., 2019; Chen et al., 2020; Gao et al., 2021; ) and *A. pisum* (Han et al., 2006; Mutti et al., 2006, 2008; Shakesby et al., 2009; Will and Vilcinskas, 2015; Vellichirammal et al., 2017; Niu et al., 2019) and CRISPR-Cas9 mutagenesis allows gene knockout in *A. pisum* (Le Trionnaire et al., 2019). Thus, the discovery of many bicycle genes in these species presents new experimental opportunities to uncover the organismal and molecular functions of *bicycle* genes.

## Materials and Methods

### Gene Structure Based Bicycle Gene Classifier

The *bicycle* gene classifier was built as a generalized linear model (GLM) with the following predictor variables: total gene length, total length of coding exons, first coding exon length, last coding exon length, mean internal exon length, and number of internal exons in phase 0, 1, and 2. All length measurements are in base pairs. All genes with zero or one internal exon were removed from the training and test datasets because mean internal exon lengths and number of internal exons starting in each of the three phases cannot be accurately estimated; hence, our classifier was unable to detect any potential *bicycle* genes with only one to three exons. The dependent variable from the GLM is the predictor of *bicycle* gene classification. The GLM was implemented with the *glm* function as *binomial* with *logit* link, and response variables for the whole-genome gene set were predicted using *predict*, both in the *R* base package *stats* v.3.6.1.

In the training set, the dependent variable of the GLM is whether the transcript was previously classified as a *bicycle* gene (Korgaonkar et al., 2021), where *bicycle* genes were coded as 1 and non-bicycle genes were coded as 0. We used only genes classified as either *bicycle* genes or previously annotated genes in the training set due to the possibility that unannotated genes include additional previously undetected *bicycle* genes. Previously annotated genes were identified as those having at least one significant match at E < 0.01 after Bonferroni correction for multiple searches with *blastp* (*blast* v.2.9.0), *blastx* (*blast* v.2.9.0), or *hmmscan* (*HMMER* v3.2.1) against the Pfam database.

We evaluated the performance of the classifier using a precision-recall curve where precision = TP/(TP + FP) and recall = TP/(TP + FN); where TP, FP, and FN are the number of true positives, false positives, and false negatives, respectively. To determine a cutoff value on the dependent variable of the GLM for *bicycle* gene classification, we tested all possible cutoffs from 0 to 0.98 in increments of 0.02 to find the cutoff resulting in a precision to recall ratio closest to 1. We first validated our model by repeatedly sampling and training on 70% of the data and testing on the remaining 30% of the data for 100 replicates and found no substantial change to the trained classifier or the resulting cutoff value (mean 0.7338, s.d. 0.1578) amongst replicates. We therefore trained the final classifier on all *H. cornu bicycle* genes and annotated non-*bicycle* genes without subsampling and determined a cutoff value of 0.72 (precision = 0.9925, recall = 0.9925). The coefficients to the independent variables in the GLM are as follows: intercept = 6.26, total exon length = −0.0206, total gene length = -0.0000642, first exon length = −0.00632, last exon length = 0.0103, mean exon length = −0.0655, number of exons in phase 0 = −3.32, phase 1 = −1.12, and phase 2 = 1.21. We evaluated the contribution of each independent variable by training the *H. cornu* dataset using GLMs with each term on its own and each term removed one at a time from the full model. We applied the GLM trained by the *H. cornu* dataset to twenty-one other species belonging to the families of Aphidoidae, Phylloxeroidae, Coccoidea, Psylloidea, Aleyrodoidea, and Fulgoroidea.

### Aphid Collections and Dissections

Aphids of *T. nigriabdominalis* were collected from galls collected on *Ulmus americana* on the grounds of Janelia Research Campus (supplementary tables S2 and S3, Supplementary Material online). Species identification was confirmed using both morphological characteristics (Walczak et al., 2019) and by comparing the mitochondrial DNA of the genome, we assembled with the mitochondrial sequences at NCBI available for many species of *Tetraneura* (Lee and Akimoto, 2015; Hebert et al., 2016). Aphids of *A. pisum* LSR1 were kindly provided by Greg Davis and grown on broad bean plants in the laboratory. Salivary glands were dissected out of multiple instars of fundatrices and their offspring for *T. nigriabdominalis* and of virginoparae for *A. pisum* and stored in 3 ul of Smart-seq2 lysis buffer (0.2% Triton X-100, 0.1 U/ml RNasin Ribonuclease Inhibitor) at −80°C for later mRNA isolation. Carcasses of both species were ground in PicoPure extraction buffer, and mRNA was prepared using the PicoPure mRNA extraction kit. RNAseq libraries were prepared as described previously (Korgaonkar et al., 2021).

### Genome Sequencing, Assembly, and Annotation of *Tetraneura nigriabdominalis*

HMW gDNA was prepared from *T. nigriabdominalis* aphids isolated from a single gall following the Salting Out Method provided by 10X Genomics (https://support.10xgenomics.com/genome-exome/sample-prep/doc/demonstrated-protocol-salting-out-method-for-dna-extraction-from-cells). 10X linked-read libraries were prepared and Chromium sequencing was performed by HudsonAlpha Institute for Biotechnology. Genomes were assembled with *Supernova* v.2.1.0 commands *run* and *mkoutput* (Weisenfeld et al., 2017).

We used 133,280,000 reads for an estimated raw genome coverage of 56.18X. The genome size of the assembled scaffolds was 344.312 Mb, and the scaffold N50 was 10 Mb. Using BUSCO completeness analysis with *gVolante* version 1.2.0 and BUSCO_v2/v3 (Nishimura et al., 2017), we found that the genome contains 1035 of 1066 (97.1%) completely detected core arthropod genes and 1043 of 1066 (97.8%) partially detected core genes. The genome was annotated for protein-coding genes using BRAKER (Hoff et al., 2019) with multiple RNA-seq libraries (supplementary table S3, Supplementary Material online).

This genome has been deposited at GenBank under the accession JAIUCT000000000.

### Differential Expression Analysis

Candidate *bicycle* genes from *H. cornu*, *T. nigriabdominalis*, and *A. pisum* identified by the classifier were manually re-annotated in Apollo (Dunn et al., 2019) guided by RNAseq data viewed in Integrative Genomics Viewer (IGV) (Thorvaldsdóttir et al., 2013). Samples used for analysis are described in supplementary table S3–S6, Supplementary Material online. Differential expression analyses were performed as described previously (Korgaonkar et al., 2021) using *edgeR* v3.28.1, and full analysis scripts are provided at https://doi.org/10.25378/janelia.17868413, https://doi.org/10.25378/janelia.17892089, and https://doi.org/10.25378/janelia.17912180 respectively.

### hmmer and BLAST Searches

The phylogeny of all species studied here was estimated using *Orthofinder* v 2.3.1 (Emms and Kelly, 2018) on the complete gene sets predicted by BRAKER for all genomes using the following settings: *-M msa -S diamond -A mafft -T fasttree*. The phylogeny was plotted using *ggtree* v. 2.0.4 in *R*.

We searched for *bicycle* homologs in all aphid species and outgroups using Position-Specific Interactive Basic Local Alignment Search Tool (PSI-BLAST) (Altschul, 1997) and *hmmer* (Eddy, 2011). We ran *PSI-BLAST* v. 2.9.0 + using *-max_target_seqs 5* and all other parameters as default. We used a cutoff of E < 0.01, Bonferroni corrected for 476 genes used as query searches. We ran profile-based *hmmsearch* v.3.2.1 using all default parameters and cutoff of E < 0.01 without multiple testing correction.

### Selection Tests

*DnDs* between *H. cornu* and *H. hamamelidis* for all transcripts in the transcriptome was calculated using the *codeml* function from the *PAML* package v4.9j (Yang, 2007). The genome-wide selection scan was performed using *SweeD-P* v.3.1 (Pavlidis et al., 2013). Details of both analyses can be found in the Methods section of Korgaonkar et al., 2021.

### Gene-Structure Aware Sequence Alignment

We performed gene structure aware alignment using *prrn5* v 5.2.0 (Gotoh, 2021) with default weighting parameters. We prepared gene-structure-informed extended fasta files that were suitable as input to *prrn5* using the *anno2gsiseq.pl* utility provided at https://github.com/ogotoh/prrn_aln/blob/master/perl/anno2gsiseq.pl. We summarized the results of these multiple sequence alignments by generating histograms with a bin size of 1 to illustrate the fraction of sequences in the alignment that had an intron at each position in the alignment.

To determine whether intron positions were shared between genes more often than expected by chance, we computed the concordance in intron positions resulting from *prrn5* alignment of groups of unrelated genes. We identified paralog groups for the whole *H. cornu* transcriptome using *Orthofinder* v 2.3.3 (Emms and Kelly, 2018) and identified three paralog groups containing at least 10 genes and having at least 10 exons for comparison with *bicycle* genes: *SLC33A1*, *abcG23*, and *nrf-6*. Intron concordance was measured as the correlation coefficient ® between the number of genes from the test gene family and the *bicycle* gene family containing an intron at each location in the alignment (supplementary fig. S16, Supplementary Material online).

### Re-Annotation of Previously Sequenced Genomes

The *P. venusta* genome is an approximately full-chromosome genome assembly that was kindly provided to us by Yiyuan Li and Nancy Moran (Li et al., 2020) prior to publication. All other genomes, except for *H. cornu* and *T. nigriabdominalis*, were downloaded from NCBI (supplementary table S5, Supplementary Material online). We downloaded RNAseq data from NCBI (supplementary table S4, Supplementary Material online) and predicted coding genes using BRAKER (Hoff et al., 2019) with the same workflow we had used previously that had allowed discovery of *bicycle* genes (Korgaonkar et al., 2021).

## Supplementary Material

Supplementary data are available at *Genome Biology and Evolution* online.

## Supplementary Material

evac069_Supplementary_Data

## Data Availability

All new sequence data generated during this study is available at NCBI at the accession numbers provided in supplementary tables S2–S4, Supplementary Material online. All analysis scripts, which allow reproduction of all analyses and reproduction of all figures, are available on Figshare with DOIs provided in supplementary table S7, Supplementary Material online. Whole transcriptome annotation files, classifier-identified *bicycle* genes lists, and multi-sequence alignment (MSA) files for all analysis where Logo plots were generated are also available on Figshare at DOIs provided in supplementary table S7, Supplementary Material online. The code for training and applying the gene-structure classifier has been instantiated as Google Colabs freely available to the public at the following URLs: https://colab.research.google.com/drive/1GtSkUDC6US1H_WVIfk3WcJUPrGKI-RY8, https://colab.research.google.com/drive/1oJAHehrX4ytqT9OZtLKPWNXQAijsixcx.
