# Supplementary material for: Gene Structure-Based Homology Search Identifies Highly Divergent Putative Effector Gene Family
Source: Genome Biol Evol. 2022 May 9;14(6):evac069. doi: 10.1093/gbe/evac069 (PMC9168663; doi:10.1093/gbe/evac069)
Supplement: evac069_Supplementary_Data [file evac069_supplementary_data.zip › Supplementary Figure Legends.pdf]

Supplementary Figures

Figure S1. Salient features of gene structure of *H. cornu* bicycle genes.

(A) Sequence similarity between *H. cornu bicycle* genes versus the distance between genes. Most of the most similar *bicycle* genes are located within 100kb of each other, supporting a model of frequent tandem or local duplication. However, many similar genes are located on different chromosomes, suggesting that *bicycle* genes often transpose to different chromosomes.

(B) The proportion of the forty flanking (20 upstream and 20 downstream) orthologous genes surrounding *bicycle* genes or randomly selected genes between *H. cornu* and *T. nigriabdominalis*. Far fewer homologous genes are found flanking *bicycle* orthologs than between randomly selected orthologs, which suggests that conserved synteny is not a highly informative measure of *bicycle* gene homology.

(C-D) Almost all internal exons of *bicycle* genes are of phase 2 (C), whereas the majority of internal exons of other genes in the *H. cornu* genome are of phase 0 (D).

(E-F) *Bicycle* genes have a wide range of exon numbers, with a distribution (E) different from non-*bicycle* genes (F).

(G-H) The total length of Bicycle proteins is better predicted by the number of exons in the gene (G) than by the length of the first and last exon (H).

Figure S2. Performance of a gene-structure based classifier of *bicycle* genes.

(A) Gene structure features used in classifier. The intron-exon structure of a generic gene is shown at the top. The six features tested in the classifier are illustrated below the gene.

(B) Precision versus recall curves for replicate training runs of the classifier using 100 different subsets of the training data. The curves across all subsets are very similar, indicating that the classifier was not unduly influenced by a specific subset of the data.

(C) Histogram of the number of transcripts with a particular classifier response shows a bimodal distribution. The vast majority of genes (>10,000) had a response close to 0. A threshold of 0.72 was chosen at a precision to recall ratio of 1.

(D) Precision (the proportion of true positives among both true and false positives) versus recall (the proportion of true positives among true positives and false negatives) for the model trained on 476 previously identified *H. cornu bicycle* genes. Differently colored dots represent the precision to recall ratio for different probability cutoffs of the model, shown in panel (D).

(E) The ratio of precision to recall for different cutoffs. Precision approximately equals recall (P/R = 1) at a cutoff of 0.72.

(F) Precision and recall values for models where one variable at a time was removed from the model. Removal of the number of exons with mode2 had the largest decrement on performance, but all models performed well.

(G) Precision and recall values for models where only one predictor variable was included in the model. Exon mean length had the best predictive value on its own, but no single variable performed as well as inclusion of all variables.

(H) Correlation amongst predictor variables for all of the original 476 *bicycle* genes. Most variables are not strongly correlated.

(I) First two principal components of principal component analysis of values for the predictor variables for all of the original 476 *bicycle* genes provides further support that variables are not strongly correlated.

(J) Percent variance explained for the eight principal components of a principal component analysis for the predictor variables for all of the original 476 *bicycle* genes.

Figure S3. Plots of exon number per gene vs median exon size for all species studied. Phylogeny for all species is shown on left and is identical to phylogeny in Figure 1. *Bicycle* genes tend to have many small exons. Red = bicycle classifier, blue = other genes.

Figure S4. Newly identified candidate *bicycle* genes in *H. cornu* exhibit signatures of strong and recent positive selection.

(A-C) d_N/d_S ratio for the 476 original *bicycle* genes (A), the newly identified putative *bicycle* genes (B) and the non-*bicycle* genomic background (C) between *H. cornu* and *H. hamamelidis*. Both the original *bicycle* genes and the new putative *bicycle* genes show an excess of genes with a ratio of d_N/d_S above 1 compared to the background. Dashed vertical line indicates d_N/d_S = 1.

(D) Median distance to the closest sweep signal for original *bicycle* genes (red dashed line) and classifier newly identified putative *bicycle* genes (blue dashed line). Histogram shows median distance to closest sweep signal for 1000 permutations of gene labels. The original *bicycle* genes and the new putative *bicycle* genes are both closer to sweep signals than expected by chance.

Figure S5. Gene-structure aware alignments reveal extensive conservation of intron locations between the originally-defined *bicycle* genes and putative *bicycle* homologs identified by the gene-structure based classifier.

(A-D) Predicted proteins encoded by the original or newly identified bicycle homologs were divided by sequence length into Unicycle (A), Bicycle (B), Tricycle (C), or Tetracycle (D)

proteins and aligned using gene-structure aware alignment. The frequency of introns at each location in the alignment is plotted for the original (orange) and newly defined (cyan) *bicycle* homologs and positions without introns were excluded from the plot. Intron positions are therefore relative to each other and do not indicate absolute position in the proteins or the alignments. Coincidence of intron positions is shown by purple bars. Original *bicycle* homologs were randomly down-sampled to 20 genes to match the number of newly identified *bicycle* homologs to improve overall alignment accuracy (B).

Figure S6. Testing intron concordance. To determine whether concordant introns were found more often than expected by chance alignment of unrelated genes, gene-structure aware alignment was performed between a subset of the original *bicycle* genes and genes from three gene families containing at least 10 genes with at least 10 exons each: *SLC33A1*, *abcG23*, and *nrf-6*. Intron concordance was measured as the correlation coefficient between the number of genes exhibiting an intron in the test gene family versus the original *bicycle* gene family.

(A) Example of gene-structure aware alignment of 23 genes from *SLC33A1* (blue) and 23 original *bicycle* gene (orange) families. One intron was found frequently in both gene families (purple), a pattern observed in most alignments and likely reflects an artifact of the gene-structure aware alignment algorithm.

(B) Example of gene-structure aware alignment of 20 candidate *H. cornu bicycle* genes identified by the classifier (blue) and 20 original *bicycle* genes (orange). Many introns are found in both groups of genes (purple).

(C-D) Examples of quantification of intron concordance, measured as the correlation between the number of genes with an intron at each location in the alignment in the test gene family versus the original *bicycle* genes. For a comparison of the unrelated gene

families *SLC33A1* and original *bicycle* genes (C), the correlation was close to 0. For a comparison of candidate *bicycle* genes versus the original *bicycle* genes, the correlation was high and positive (D).

(E) Intron concordance—measured as the correlation coefficient (*R*) of the number of genes sharing intron locations in gene-structure aware alignments—of repeated re-samplings of unrelated genes families generated a null distribution of expected *R* values for unrelated genes (*SLC33A1*, *abcG23*, and *nrf-6* versus original *bicycle* genes in red, green and blue histograms, respectively). *R* values for all comparisons of candidate *bicycle* genes found by the classifier and the original *bicycle* genes were all positive and much higher than any of the *R* values resulting from alignment of unrelated gene families, indicating that more introns are shared between genes in these gene-structure aware alignments than expected between unrelated gene families by chance (P < 0.01).

Figure S7. *Tetraneura nigriabdominalis* life cycle and additional features of *T. nigriabdominalis bicycle* genes.

(A) Photo of mature galls of *T. nigriabdominalis* on leaf of *Ulmus americana* collected at Janelia Research Campus, Ashburn, VA, USA.

(A) Diagram of life cycle of *T. nigriabdominalis*. Samples for differential expression analysis were collected from generations G1 and G2.

(B) The *T. nigriabdominalis* putative bicycle homologs from Clusters 1 and 4, with and without CYC motifs, respectively, share multiple intron positions. Proteins from the two classes of genes were aligned using gene-structure aware alignment and the frequency of introns at each location in the alignment was plotted in different colors for the two classes of genes and positions without introns were excluded from the plot. Intron

positions are therefore relative to each other and do not indicate absolute position in the proteins or the alignments. Sites of coincident introns are indicated in purple.

Figure S8. The *A. pisum* putative *bicycle* homologs share multiple intron positions with *H. cornu bicycle* genes. Proteins were aligned using gene-structure aware alignment and the frequency of introns at each location in the alignment was plotted in different colors for the two classes of genes and positions without introns were excluded from the plot. Intron positions are therefore relative to each other and do not indicate absolute position in the proteins or the alignments. Sites of coincident introns are indicated in purple.

Figure S9. Features of *bicycle* homologs detected in aphid species.

(A-B) The number of *bicycle* homologs detected by the gene-structure based classifier versus are not correlated with genome N50 (A; R^2 = 0.09, F = 1.4, P = 0.26) nor with the number of genes annotated for each genome (B; R^2 = 0.002, F = 0.4, P = 0.69).

(C-F) Logo plots of putative *bicycle* homologs detected in four aphid species, *M. persicae* (C), *P. nigronervosa* (D), *R. maidis* (E), and *C. cedri* (F) reveals that they all contain CYC motifs. The *M. persicae* and *P nigronervosa* genes are primarily *unicycles*, the *R. maidis* genes are primarily *bicycles*, and the *C. cedri* genes are primarily *tetracycles*.

Figure S10. Features of *bicycle* homologs detected in outgroups to aphids.

(A-C) Logo plots of proteins encoded by putative *bicycle* homologs detected in *D. vitilifoliae* (A), *M. hirsutus* (B), and *P. solenopsis* (C). The logo plots from these putative homologs show no obvious CYC motifs.

(D-F) Gene-structure aware alignments reveal that multiple introns are shared between these highly divergent putative *bicycle* homologs and *H. cornu bicycle* genes. Positions

in alignments without introns were excluded from the plot. Intron positions are therefore relative to each other and do not indicate absolute position in the proteins or the alignments. Sites of coincident introns are indicated in purple.

Figure S11. *Megacycle* genes share intron locations with *bicycle* genes and are found in many aphid species.

(A) Gene-structure aware alignments reveal that multiple introns are shared between *megacycle* genes and *H. cornu bicycle* genes. Positions in alignments without introns were excluded from the plot. Intron positions are therefore relative to each other and do not indicate absolute position in the proteins or the alignments. Shared introns are shown in purple.

(B) Maximum likelihood phylogeny of the protein sequences encoded by *megacycle* genes found in aphid species. *Megacycle* genes were not detected outside of aphids. The phylogeny has a topology similar to the aphid portion of the whole-proteome phylogeny shown in Figure 1. Only one *megacycle* gene was detected in most aphid species, but three paralogs were detected in *C. cedri* and *T. nigriabdominalis*. Values at nodes are bootstrap support values. Scale bar is 1 substitution per residue.
