# Supplementary material for: Gene Structure-Based Homology Search Identifies Highly Divergent Putative Effector Gene Family
Source: Genome Biol Evol. 2022 May 9;14(6):evac069. doi: 10.1093/gbe/evac069 (PMC9168663; doi:10.1093/gbe/evac069)
Supplement: evac069_Supplementary_Data [file evac069_supplementary_data.zip › Supplementary Tables.pdf]

Supplementary Tables

Table S1. Many microexon genes producing secreted putative effector proteins in *A. pisum* detected by mass spectrometry of secreted proteins (Dommel et al., 2020) are identified as *bicycle* genes by the classifier.

**Gene Name (Dommel)**	**Transcript ID**	**Gene Locus**	**Best hit Augustus gene**	**Peptide sequence**	***bicycle* gene according to classifier**	**Notes**
KQY1a, b	NM_001162442, AK339882	LOC100158789	g118916	MIFFKQYSMMITFIVIAVWVMPAITSE	Yes	
KQY2a, b	AK342599 & AK342927	LOC100302371 & LOC100302371	g2591	MVFYKQYLLTITCIVITAWVIPTSA	Yes	
KQY3	AK341661	LOC100301916	g48042	MVFFKQYLITLTCIVISVWITPVNT	Yes	
KQY4a, b, c, d	AK342948, AK342242, AK342690, AK342678	LOC100302370	g139706	MIFFKQYLIILTFIVIAVLVMPVTP	Yes	
KQY5a, b	AK342406, AK342378	LOC100302375, LOC100302376	g108486a	MVFFKQYLLTLTCIVIVVQVMPASA	Yes	
KQY6	AK340126	LOC100302481	g119690 and g3762	MIFFKQYLIMLTFIIIAVWVMPANT	Yes	
KQY7	AK340563	LOC100302485	g103944	MSFFKQYLTLTFIVISVWNMSEA	Yes	
KQY8	AK342473	LOC100302439	g123349	MVFFKQFLITLTVIIITEA	?	
KQY9	AK342683	LOC100302403	No close matches	MVFFKLYLLTLTCIVIAVWVMPVSA	No	Closest match, 76% identity to g2591
KQY10	AK342808	LOC100302381	g39312	MFNVLIILSLISYTFEPSYTLYKFKMVFFKQDLLMLTCITIAVWIMPPSASTN	Yes	
KQY11	AK340121	LOC100302480	g144677	MVFFRQFLITLSVILITEA	No	
KQYMp	XM_022322515	LOC111039170	g115805	MHFFKHYLIVLTYIVISFWFMPSASL	Yes	Myzus persicae
KHI1a, b	AK339863, AK339862	LOC100159750	g119277	MFKHIIVLVLCFMAYFVGNLDA	Yes	85% identical
KHI2a, b, c	AK341162, AK341161, AK342769	LOC100302383	g101192	MDKHIIMLALCLMVYIIGNIDA	Yes	
KHI3a, b	AK340197, AK342603	LOC100166702	g134738	MLKHIIVLALYLMAYIIGNIDA	Yes	
KHI4	AK341077	LOC100570519	g141770	MLKHILLALCFMAYIIENIG	Yes	
KHI5	AK341390	LOC100534636	?	MLKHILLALCFMAYIIENIGA		g141770 closest match, but no match to terminal A.
KHI6	AK340760	LOC100571631	g121945	MLKHIIVLVLCFMPYIIG	Yes	94% identical

C002Ap	XM_001948323	LOC100167863	g50444	MGSYKLYVAVMAIAIAVVQEVRC	No	2 exon gene; not searched by classifier

Table S2—Details of *Tetraneura nigriabdominalis* sample, sequence files, and SRA accession numbers for genome sequencing. JRC refers to Janelia Research Campus, Ashburn VA, USA. These sequences can be found under the SRA BioProject PRJNA759586.

**Collection Location**	**Species**	**Library Prep Method**	**Sequencing Method**	**# Read pairs**	**Accession #**
JRC	*Tetraneura nigriabdominalis*	10X Chromium	Illumina PE151	441,975,717	SRR15704183

Table S3 - Details of biological samples, sequence files, and SRA accession numbers for *Tetraneua nigriabdominalis* RNA-sequencing. JRC refers to Janelia Research Campus, Ashburn VA. These sequences can be found under the SRA BioProject PRJNA762862.

Sample ID	Collection Date	Collection Location	Generation	Organ	Library Prep Method	Sequencing Method	# Reads	Accession #
Tnig_Fund_SG_2	4/22/19	JRC	1	SG	WGB	Illumina PE150	8475508, 5308849	SRR18396612
Tnig_Fund_SG_4	4/22/19	JRC	1	SG	WGB	Illumina PE150	6087930, 6250323	SRR18396611
Tnig_Fund_SG_5	4/22/19	JRC	1	SG	WGB	Illumina PE150	7474183, 7147731	SRR18396610
Tnig_Fund_Carc_2	4/22/19	JRC	1	Carcass	WGB	Illumina PE150	6208251, 6194859	SRR18396609
Tnig_Fund_Carc_4	4/22/19	JRC	1	Carcass	WGB	Illumina PE150	7453030, 6095776	SRR18396608
Tnig_Fund_Carc_5	4/22/19	JRC	1	Carcass	WGB	Illumina PE150	8399343, 7332616	SRR18396607
Tnig_2ndGen_SG_2_1	4/22/19	JRC	2	SG	WGB	Illumina PE150	6902051, 7037142	SRR18396606
Tnig_2ndGen_SG_2_2	4/22/19	JRC	2	SG	WGB	Illumina PE150	6888742, 6997123	SRR18396605
Tnig_2ndGen_SG_2_3	4/22/19	JRC	2	SG	WGB	Illumina PE150	6049169, 6696157	SRR18396604

Table S4 - Details of biological samples, sequence files, and SRA accession numbers for *Acyrthosiphon pisum* RNA-sequencing. The aphid strain used was LSR1 (Caillaud et al., 2002), originally collected in the vicinity of Ithaca, NY, USA in summer 1998. Aphids were grown on broad bean plants in the laboratory at Janelia Reseach Campus and dissected for these samples on 21 February 2021. Sequences can be found under SRA BioProject PRJNA762703.

Sample ID	Collection Date	Collection Location	Generation	Organ	Library Prep Method	Sequencing Method	# Reads	Accession #
SG1	1998	Ithaca, NY, USA	1	SG	WGB	Illumina PE150	1273082, 5724222	SRR15862168
SG2	1998	Ithaca, NY, USA	1	SG	WGB	Illumina PE150	1557673, 6606339	SRR15862167
SG3	1998	Ithaca, NY, USA	1	SG	WGB	Illumina PE150	1199959, 5221105	SRR15862166
SG4	1998	Ithaca, NY, USA	1	SG	WGB	Illumina PE150	1159521, 5169430	SRR15862165
carc1	1998	Ithaca, NY, USA	1	Carcass	WGB	Illumina PE150	998879, 4363153	SRR15862164
carc2	1998	Ithaca, NY, USA	1	Carcass	WGB	Illumina PE150	1185062, 5433749	SRR15862163
carc3	1998	Ithaca, NY, USA	1	Carcass	WGB	Illumina PE150	1045749, 4901408	SRR15862162

Table S5 – SRA accession numbers for Genomes downloaded from NCBI. Manually corrected BRAKER annotations are available on Figshare at 10.25378/janelia.17777888.

Species	Original genomes Genbank assembly accession or alternative location	Publication source of each genome	Manually corrected BRAKER Annotation	TRINITY assembly
Acyrthosiphon pisum	https://bipaa.genouest.org/sp/acyrthosiphon_pisum/download/genome/JIC1_1.0/	(Mathers et al., 2021)	Apis.Augustus.updated_w_annots.22xii21.gff3	apis.Trinity.fasta.gz
Aphis gossypi	GCF_004010815.1	(Quan et al., 2019)	Agos.Augustus.updated_w_ annots.6ix21.gff3	agos.Trinity.fasta.gz
Bemisia tabaci	GCA_001854935.1	(Chen et al., 2016)	Btab.Augustus.updated_w_ annots.7vii21.gff3	
Cinara cedri	GCA_902439185.1	(Julca et al., 2020)	Cced.Augustus.updated_w_ annots.7vii21.gff3	cced.Trinity.fasta.gz
Daktulosphaira vitifoliae	https://bipaa.genouest.org/sp/daktulosphaira_vitifoliae/download/genome/v3.1/Dv_mitochondrial_genome.fa	(Rispe et al., 2020)	Dvit.Augustus.updated_w_ annots. 6ix21.gff3	
Diaphorina citri	GCA_000475195.1	(Saha et al., 2017)	Dcit.Augustus.updated_w_ annots.7vii21.gff3	
Diuraphis noxia	GCA_001186385.1	(Nicholson et al., 2015) (Zhang et al., 2014)	Dnox.Augustus.updated_w_ annots.7vii21.gff3	dnox.Trinity.fasta.gz
Ericerus pela	GCA_011428145.1	(Yang et al., 2019)	Epel.Augustus.updated_w_ annots.7vii21.gff3	
Eriosoma lanigerum	GCA_013282895.1	(Biello et al., 2021)	Elan.Augustus.updated_w_ annots.7vii21.gff3	elan.Trinity.fasta.gz
Hormaphis cornu	GCA_017140985.1	(Korgaonkar et al., 2021)	Augustus.updated_w_ annots.19viii21_final.gff3	hcor.Trinity.fasta.gz

Laodelphax striatella	GCA_003335185.2	(Zhu et al., 2017)	Lstr.Augustus.updated_w_ annots.7vii21.gff3	
Maconellicoccus hirsutus	GCA_003261595.1	(Kohli et al., 2021)	Mhir.Augustus.updated_w_ annots. 6ix21.gff3	
Melanaphis sacchari	GCA_002803265.2	https://data.nal.usda.gov/dataset/melanaphis-sacchari-strainlsu-and-endosymbiont-genome-sequencing-and-assembly	Msac.Augustus.updated_w_ annots.7vii21.gff3	msac.Trinity.fasta.gz
Myzus persicae	https://bipaa.genouest.org/sp/myzus_persicae/download/genome/v2.0/	(Mathers et al., 2021)	Mper.Augustus.updated_w_annots.13xii21.gff3	mper.Trinity.fasta.gz
Nilaparvata lugens	GCA_000757685.1	(Xue et al., 2014)	Nlug.Augustus.updated_w_ annots. 6ix21.gff3	
Pachypsylla venusta	GCA_012654025.1	(Y. Li et al., 2020)	Pven.Augustus.updated_w_ annots.7vii21.gff3	
Pentalonia nigronervosa	GCA_014851325.1	(Mathers et al., 2020)	Pnig.Augustus.updated_w_ annots. 6ix21.gff3	pnig.Trinity.fasta.gz
Phenacoccus solenopsis	GCA_009761765.1	(M. Li et al., 2020)	Psol.Augustus.updated_w_ annots. 6ix21.gff3	
Rhopalosiphum maidis	GCF_003676215.2	(Chen et al., 2019)	Rmai.Augustus.updated_w_ annots. 6ix21.gff3	rmai.Trinity.fasta.gz
Sipha flava	GCA_003268045.1	https://data.nal.usda.gov/dataset/crop-rotational-diversity-affects-soil-microbiomes	Sfla.Augustus.updated_w_ annots.6ix21.gff3	sfla.Trinity.fasta.gz
Tetraneura nigriabdominalis	JAIUCT000000000	NA	Tnig.Augustus.updated_w_ annots.7vii21.gff3	Tnig.Trinity.fasta.gz
Trialeurodes vaporariorum	GCA_011764245.1	(Xie et al., 2020)	Tvap.Augustus.updated_w_ annots.7vii21.gff3	

Table S6 – SRA accession numbers for RNAseq samples downloaded from NCBI. See separate file “S6_RNAseq_SRA_accessions.xlsx”.

Table S7 – Figshare DOIs of manually corrected BRAKER files and bicycle gene lists for all twenty-two species, and all coding scripts. Available as a single Collection at https://doi.org/10.25378/janelia.c.5778905.

**Item name**	**Figshare DOI**
Whole transcriptome annotation files	10.25378/janelia.17777888
Classifier-identified *bicycle* genes lists	10.25378/janelia.17783336
Multi-sequence alignment files for all analysis in this manuscript where Logo plots were generated from MSAs	10.25378/janelia.19560991
Sequence-based identification of *bicycle* genes scripts	10.25378/janelia.17864489
Gene structure of *H. cornu bicycle* genes script	10.25378/janelia.17845784
Linear regression classifier development and application scripts	10.25378/janelia.17841809

H. cornu classifier-identified *bicycle* genes expression scripts	10.25378/janelia.17868413
Selection signatures in classifier-identified *bicycle* genes scripts	10.25378/janelia.17846639
T. nigriabdominalis classifier-identified *bicycle* genes expression scripts	10.25378/janelia.17892089
A. pisum classifier-identified *bicycle* genes expression scripts	10.25378/janelia.17912180
bicycle genes identification in outgroup species scripts	10.25378/janelia.17912189
prrn5 gene structure aware alignment scripts	10.25378/janelia.17936483
