# Supplementary figures and images for: Gene Structure-Based Homology Search Identifies Highly Divergent Putative Effector Gene Family
Source: Genome Biol Evol. 2022 May 9;14(6):evac069. doi: 10.1093/gbe/evac069 (PMC9168663; doi:10.1093/gbe/evac069)

Figure S1

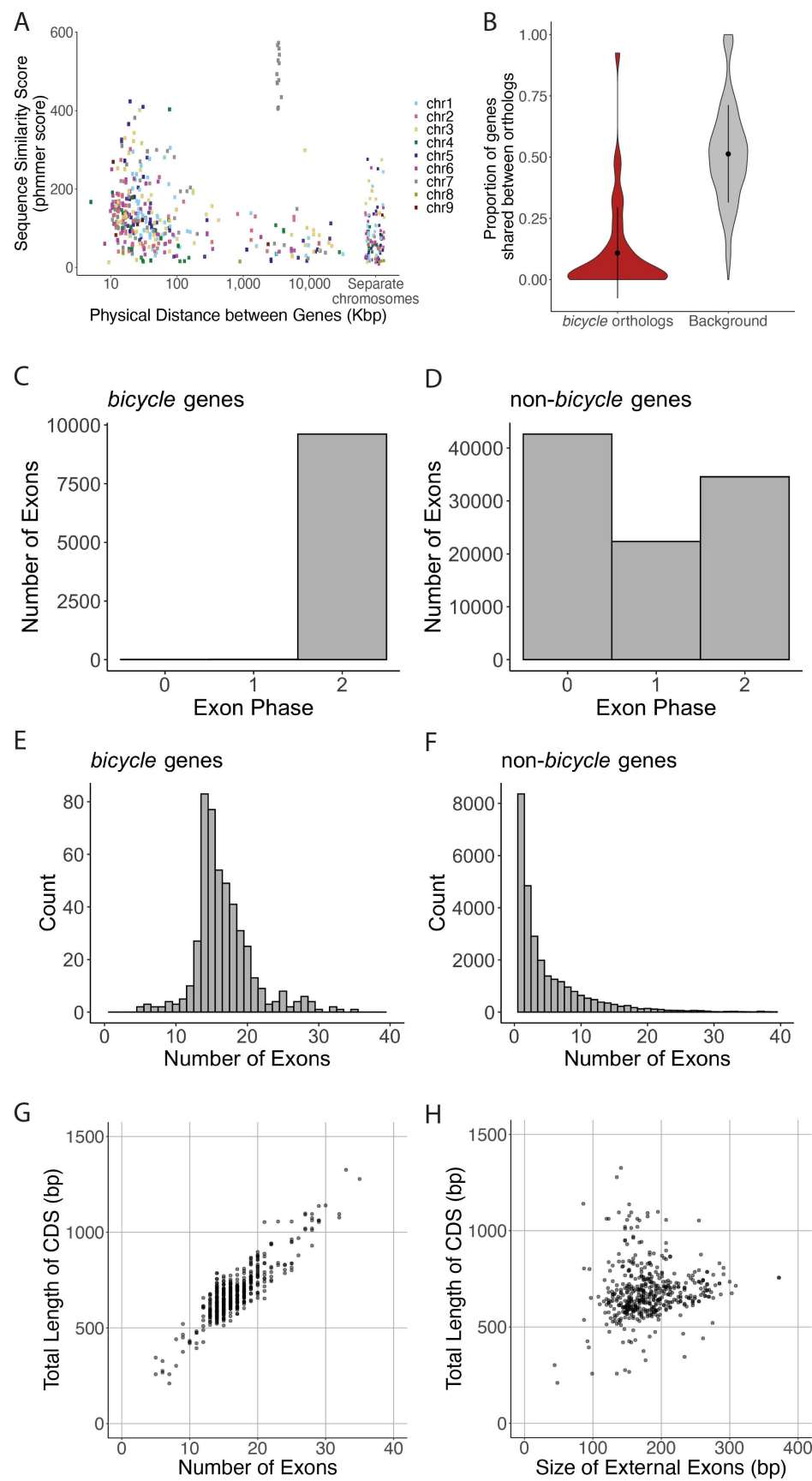

Figure S2

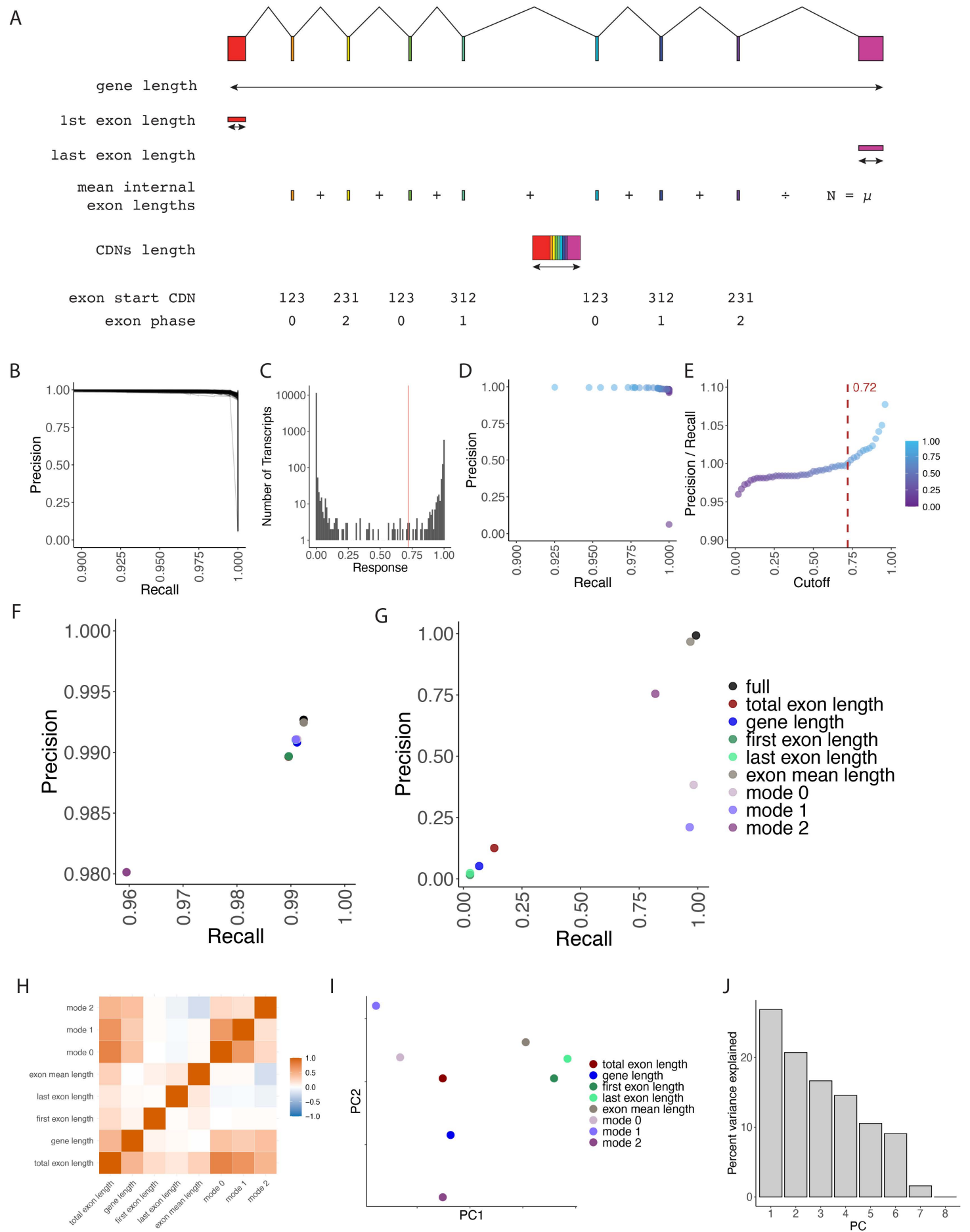

Figure S3

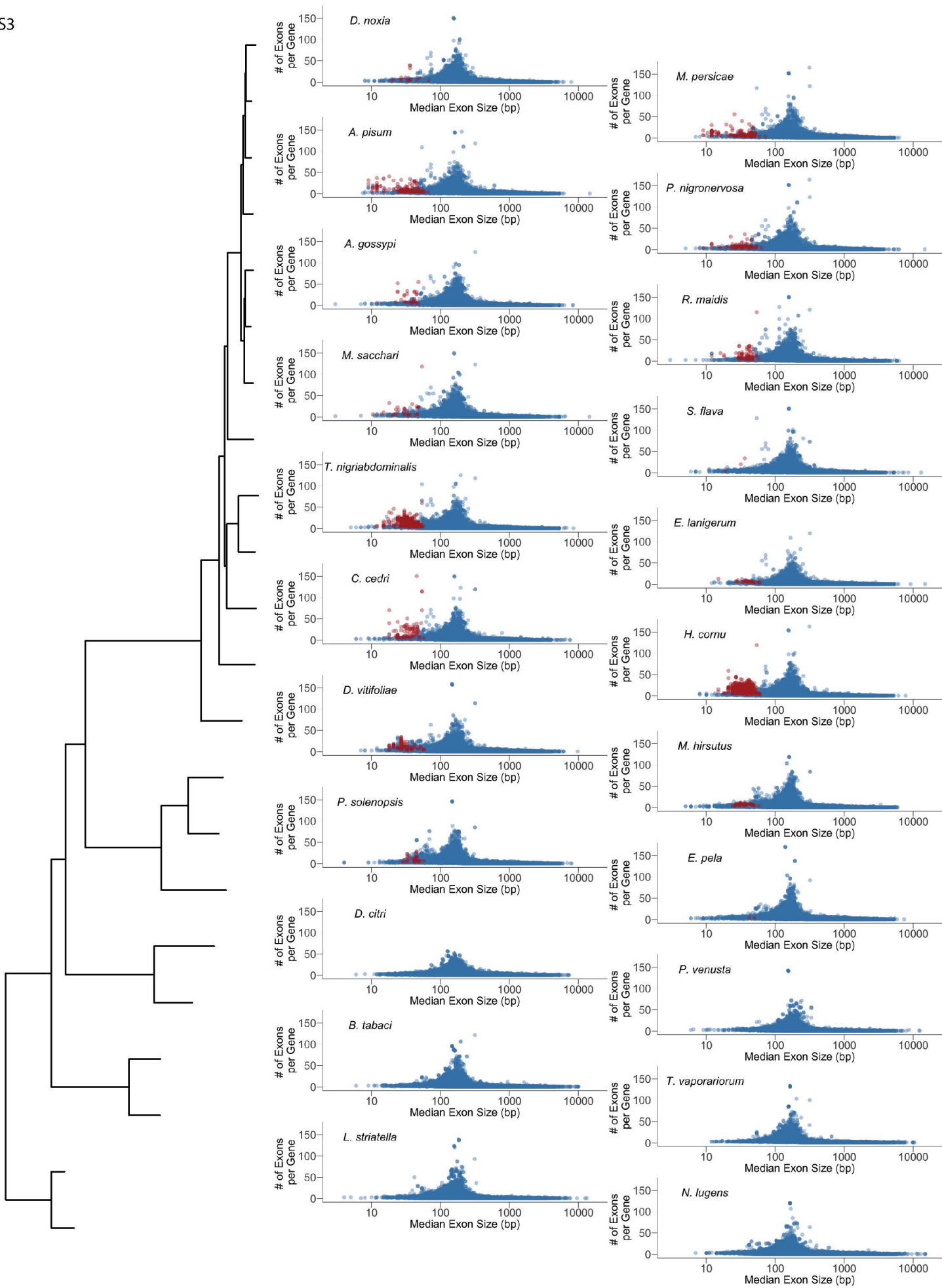

Figure S4

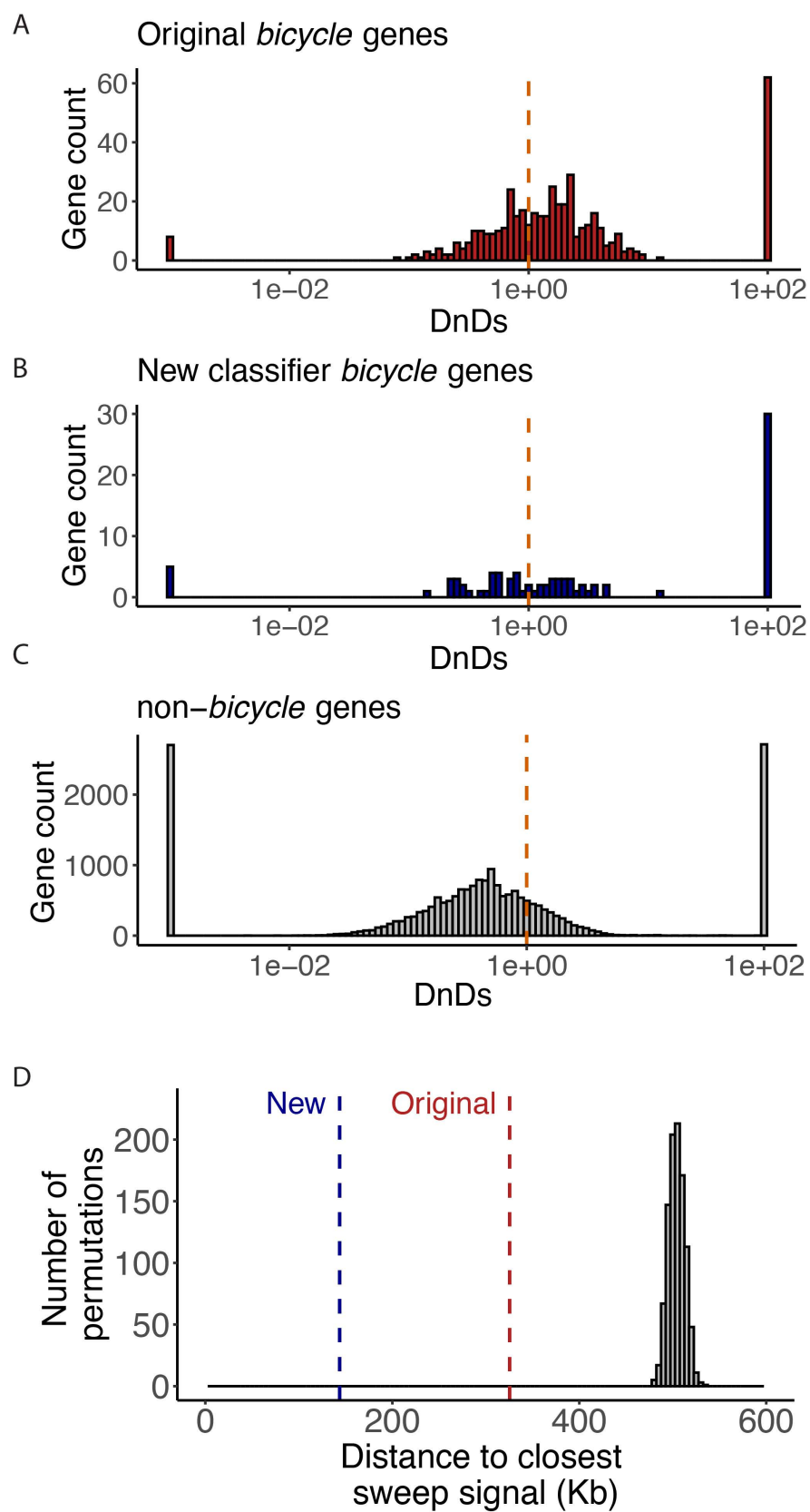

Figure S5

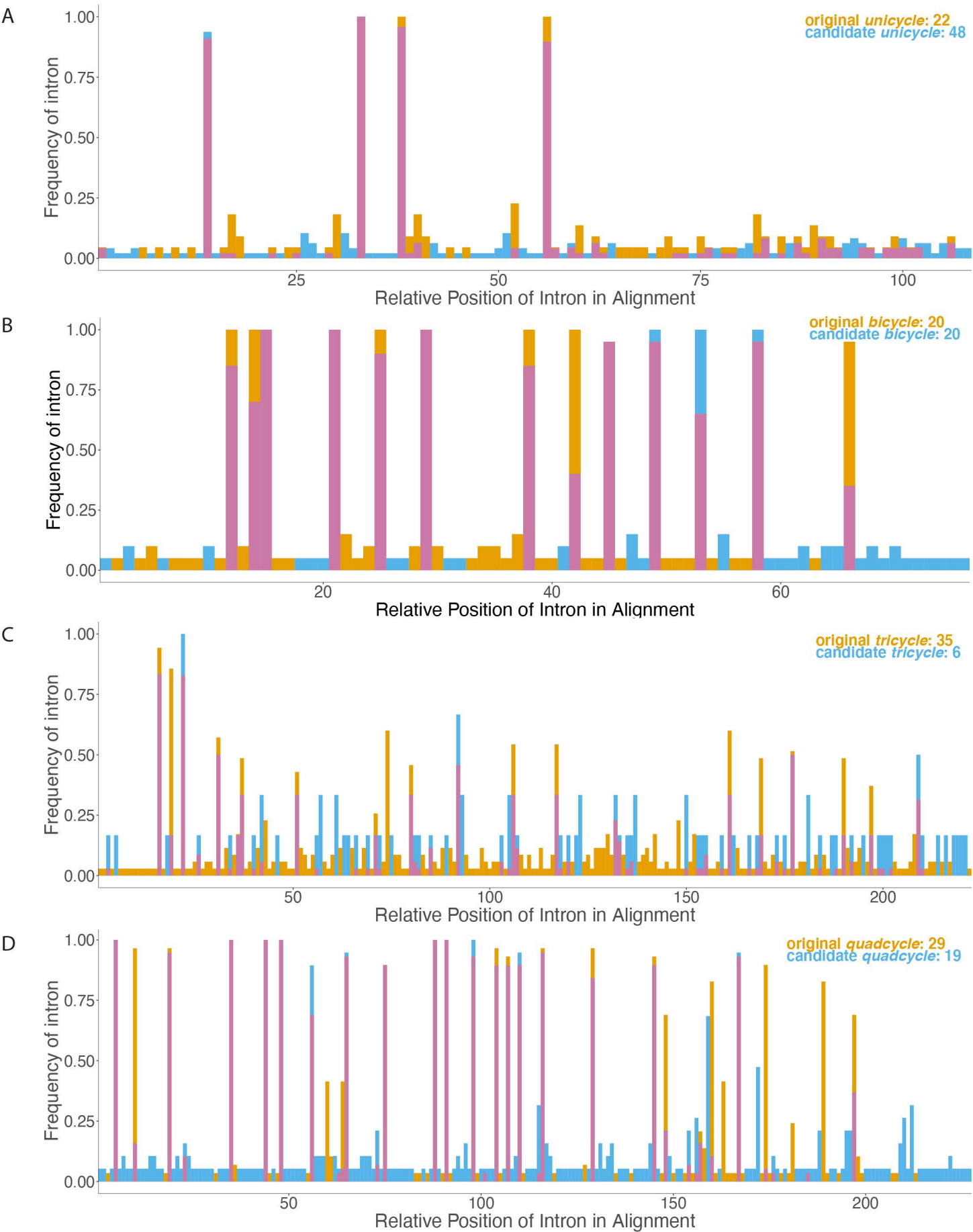

Figure S6

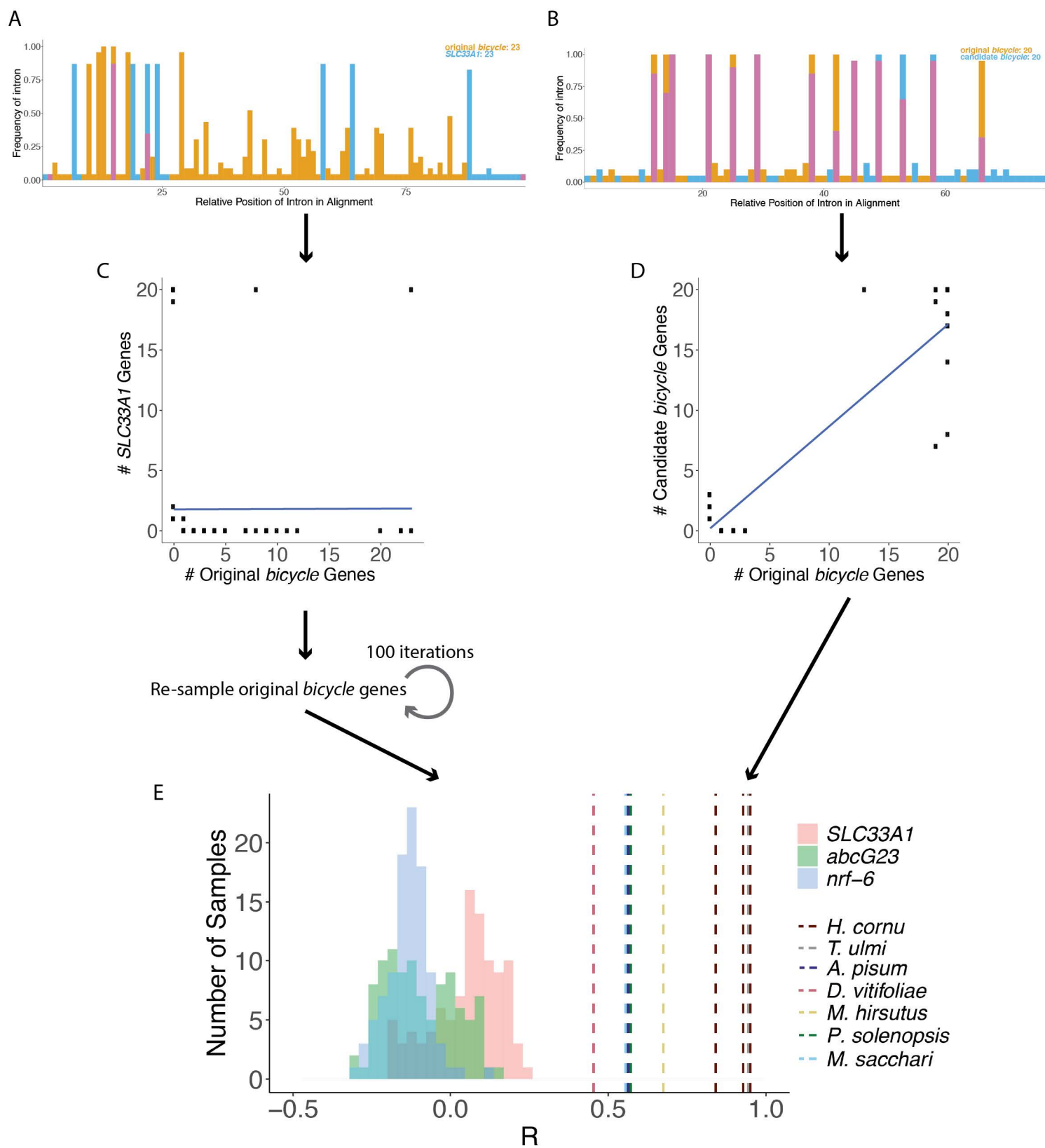

Figure S7

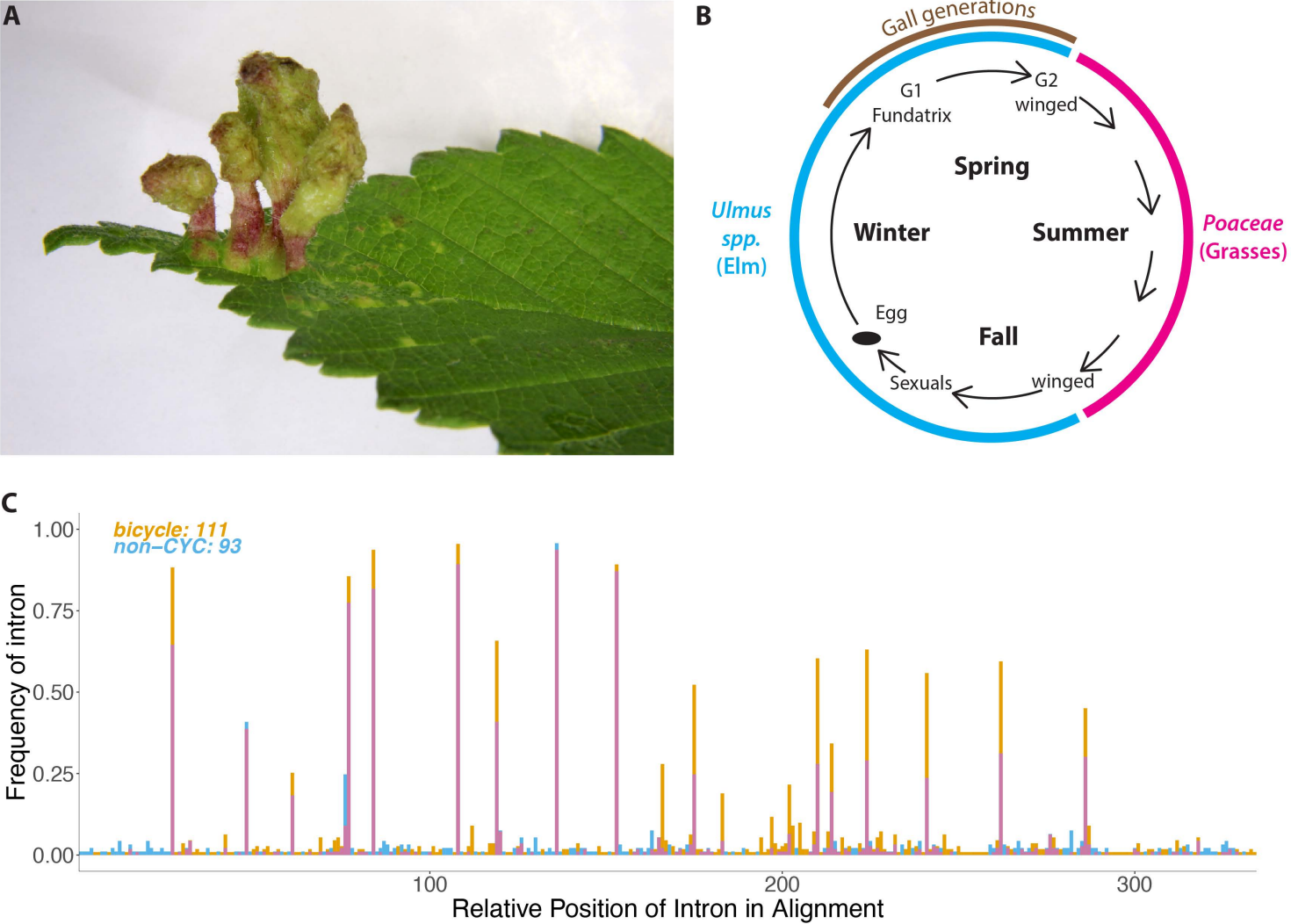

Figure S8

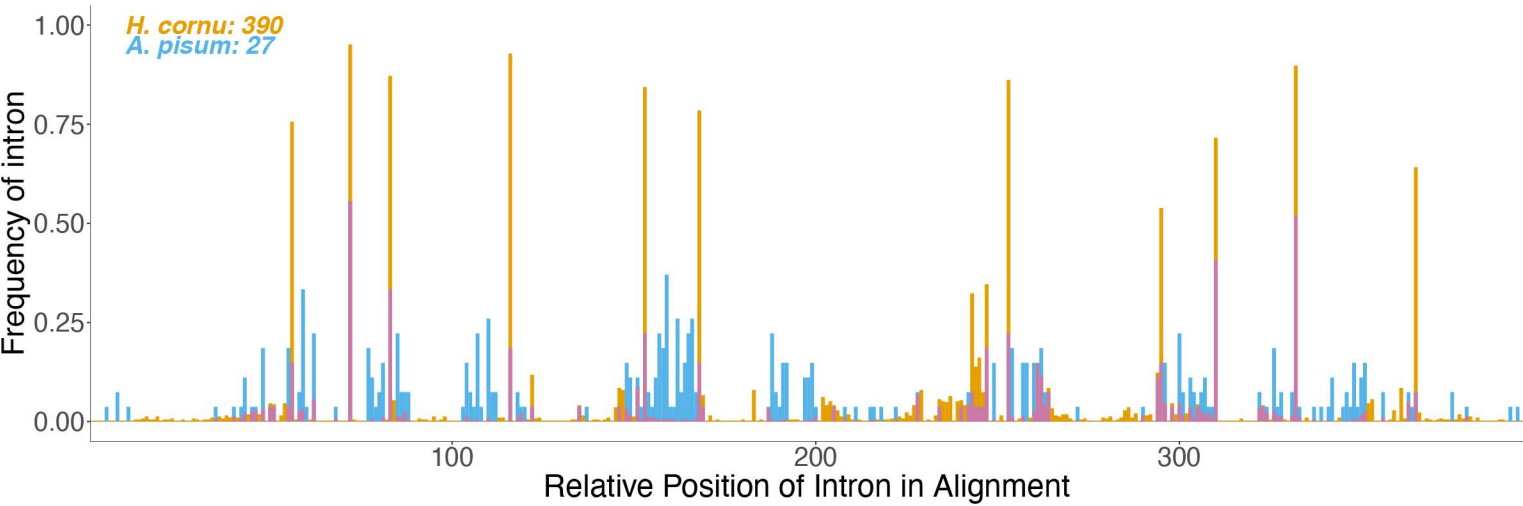

Figure S9

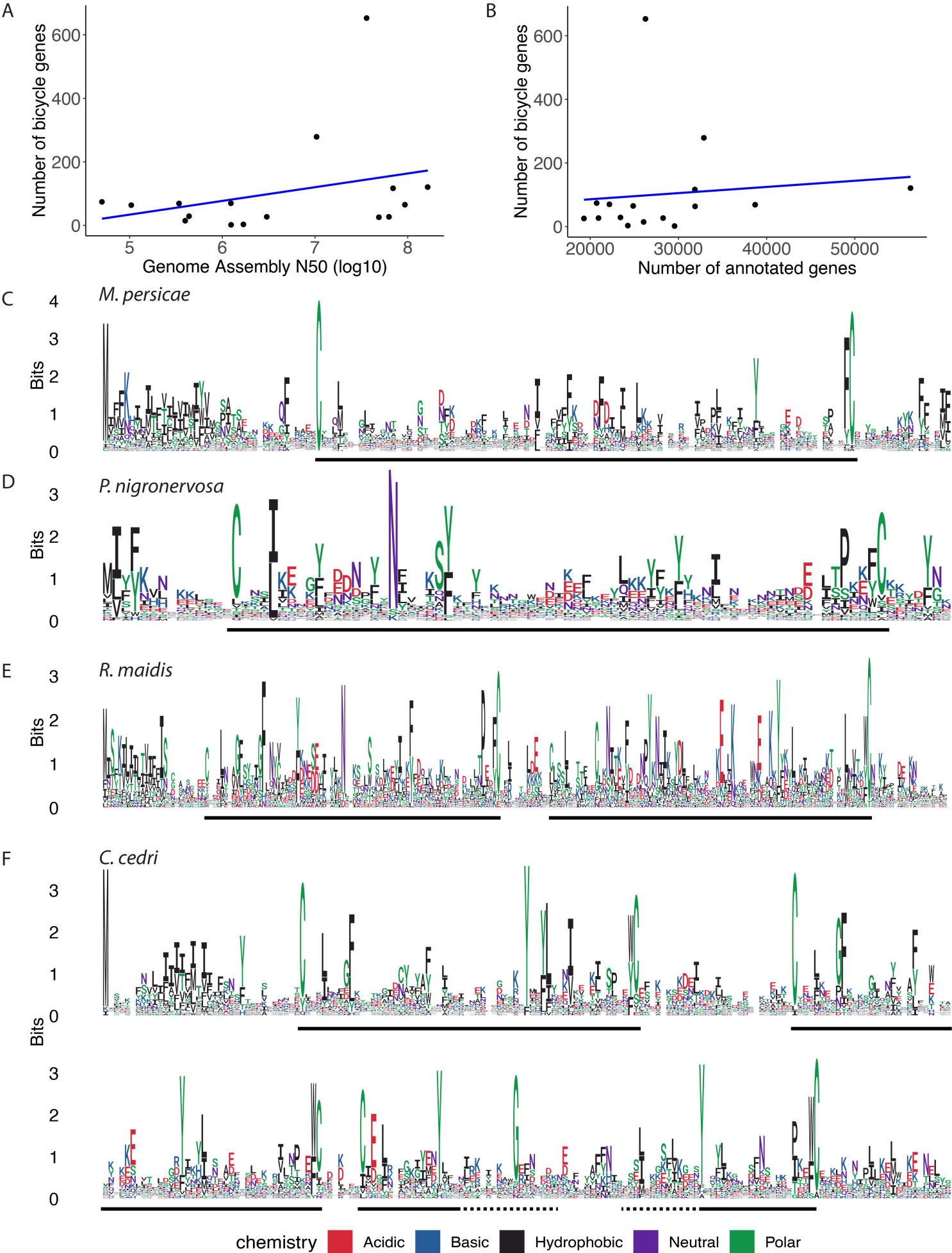

Figure S10

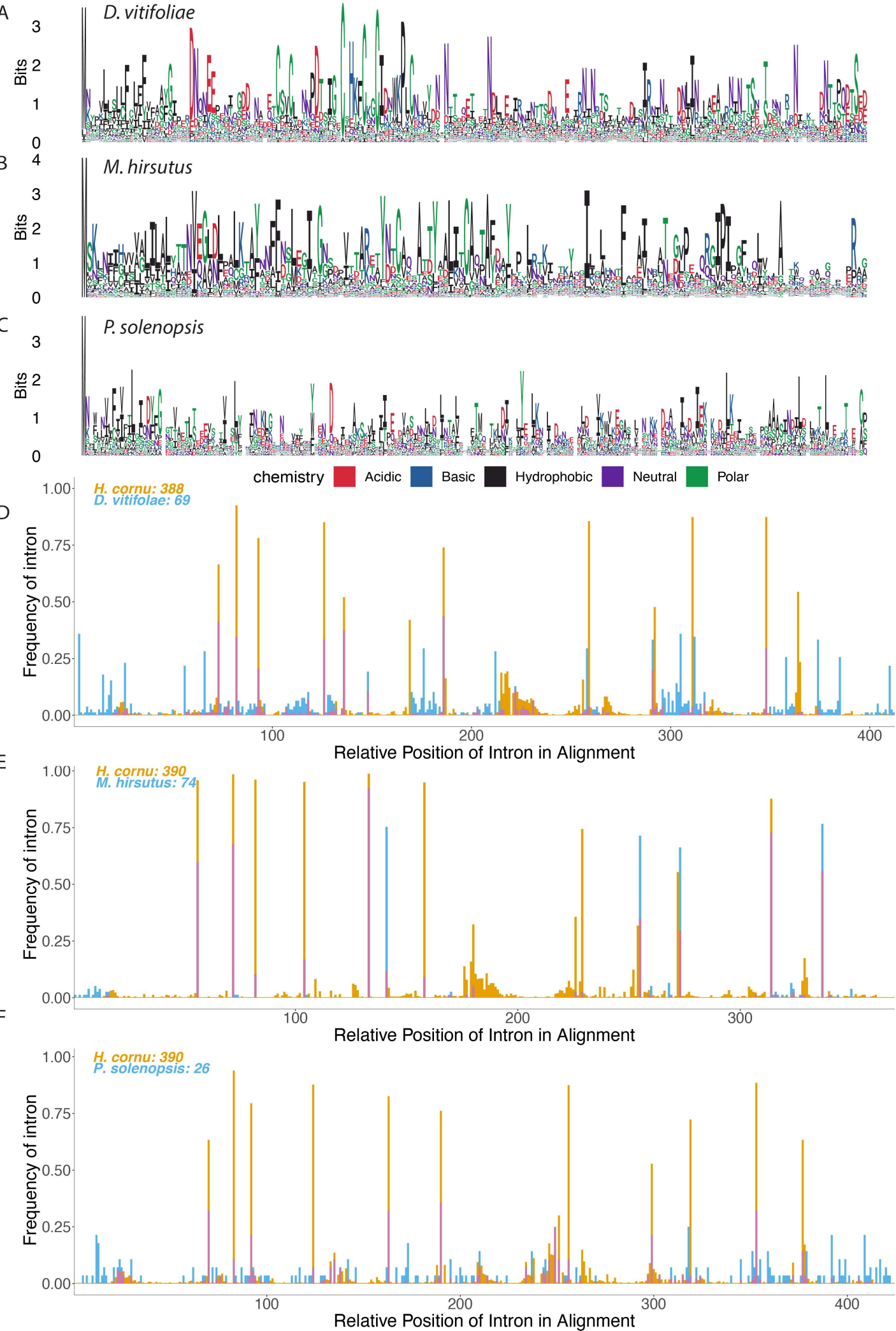

Figure S11

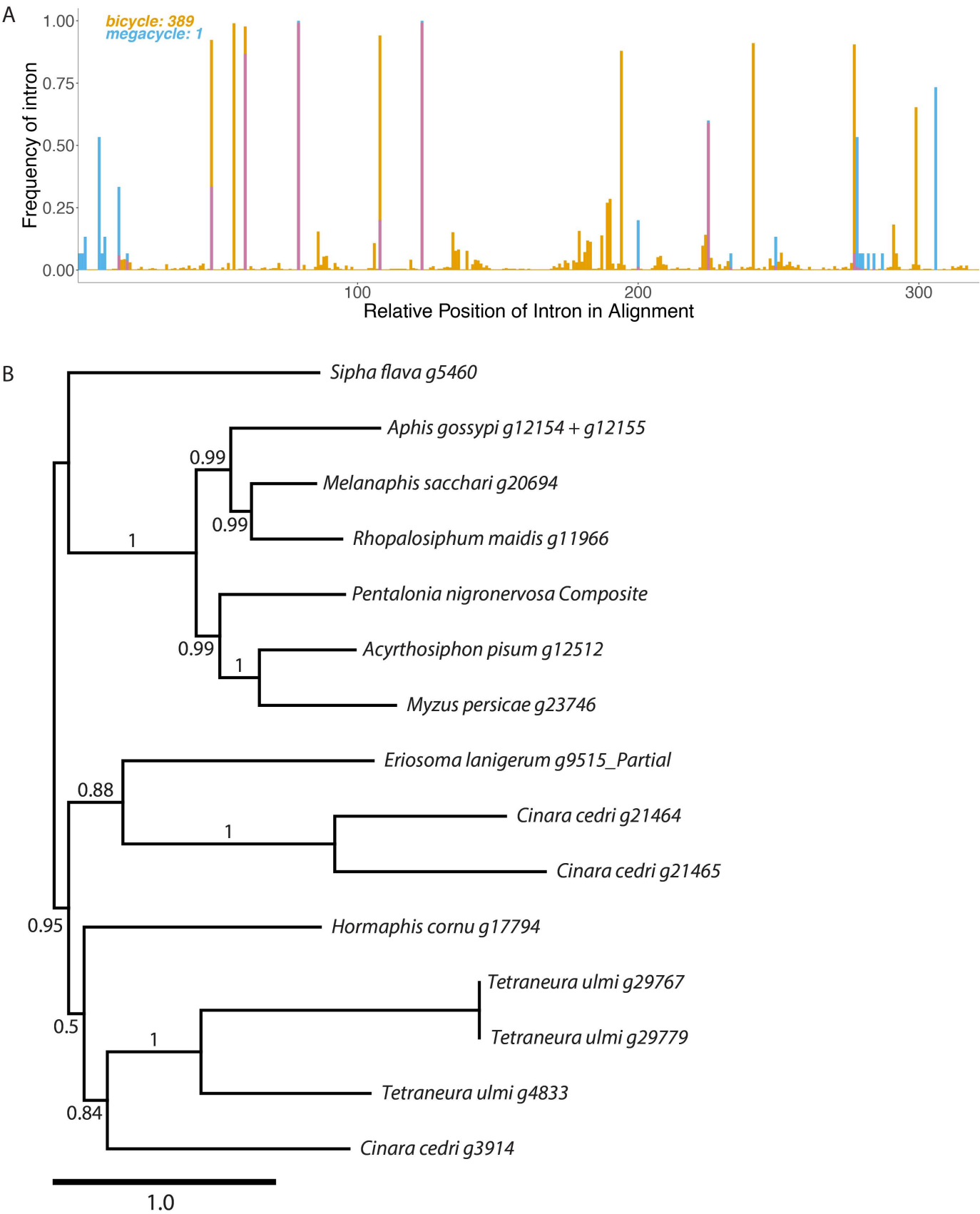

Supplement: evac069_Supplementary_Data [file evac069_supplementary_data.zip › Figures_supplementary.pdf]
